# Integrative Analysis of lncRNA–RBP (RNA-Binding Protein) Regulatory Networks Reveals Molecular Targets for Enhancing *Zea mays* Resistance to *Aspergillus flavus* and Aflatoxin Contamination

**DOI:** 10.3390/ijms27052493

**Published:** 2026-03-08

**Authors:** Ramya Parakkunnel, Bhojaraja Naik Keshava, Manjanagouda Siddanagouda Sannagoudar, Samudrala Prashant Jeevan Kumar, Kuldip Jayaswall, Aravindan Sundaram, Anandan Annamalai

**Affiliations:** 1 ICAR-National Institute of Seed Science and Technology (ICAR-NISST), Regional Station, GKVK Campus, Bengaluru 560065, India; 2 ICAR-Directorate of Floricultural Research, Pune 411036, India; 3 ICAR-National Institute of Seed Science and Technology (ICAR-NISST), Mau 275103, India

**Keywords:** *Aspergillus flavus*, aflatoxin, lncRNA, *Zea mays*, machine learning, molecular docking, RNA binding proteins

## Abstract

*Aspergillus flavus* infection and accumulation of carcinogenic aflatoxins are detrimental to maize (*Zea mays*) production and consumption. We investigated lncRNA–RBP interactions during maize–*A. flavus* crosstalk using transcriptomic profiling, structural analysis, molecular docking simulations, and machine learning approaches. Analysis of 18 RNA-seq datasets identified 2104 lncRNAs in maize, of which 461 were differentially expressed under *A. flavus* infection. Distinct lncRNAs were preferentially induced under infection (e.g., *Zm00001eb303170*) or normal germination (e.g., *Zm00001eb144150*, *Zm00001eb406410*). RNA secondary structure predictions indicated high structural heterogeneity and thermodynamic stability, consistent with dynamic regulatory potential. Docking simulations with six key RNA binding proteins (RBPs)—including branch point bridging protein (BPB), KH domain protein, and pentatricopeptide repeat (PPR) proteins—demonstrated strong lncRNA–protein binding, with the lncRNA1–BPB complex exhibiting the highest binding affinity. ML algorithms identified the crucial role of tryptophan in determining interactions, while lncRNA17-KH and lncRNA1-BP complexes were found to have the best interaction under normal germination and *A. flavus* infection, respectively. The lncRNA–miRNA–mRNA regulatory network highlighted lncRNAs functioning as decoys or precursors of stress-responsive miRNAs (e.g., zma-miR156, zma-miR164, zma-miR399). These interactions targeted transcriptional regulators, splicing factors, and metabolic enzymes implicated in stress tolerance, seed germination, and systemic acquired resistance. The maize lncRNAs are active regulatory molecules embedded in complex RBP and miRNA interaction networks that fine-tune gene expression during *A. flavus* infection. The study provides novel insights into lncRNA-mediated resistance mechanisms and offers potential molecular targets for breeding or gene editing to mitigate aflatoxin contamination.

## 1. Introduction

Due to their immobile nature, plants have evolved intricate defense and adaptation mechanisms to cope with biotic (e.g., pathogens, herbivores) and abiotic (e.g., drought, salinity, temperature extremes) stresses [1]. They respond to environmental changes through complex mechanisms involving stress signaling, transcriptional and post-transcriptional regulation, epigenetic modifications, and physiological and metabolic adjustments. Stress perception begins with specialized receptors that activate signaling pathways mediated by phytohormones such as abscisic acid, salicylic acid, and jasmonic acid [2]. Transcriptional regulation involves stress-responsive transcription factors like NAC, HSF, WRKY, AP2-ERF (DREB), and MYB, which modulate gene expression to coordinate stress responses [3]. Additionally, plants employ epigenetic mechanisms such as DNA methylation, histone modifications, and chromatin remodeling to establish stress memory, enhancing their ability to withstand recurring stress conditions [4]. Beyond these innate defense strategies, plants mitigate stress through antibiosis, antioxidant production, and secondary metabolite synthesis, which drive physiological and metabolic changes [5]. Recent studies highlight the crucial role of post-transcriptional modifications, particularly those mediated by long non-coding RNAs (lncRNAs) and RNA-binding proteins, in fine-tuning gene expression and optimizing spatial and temporal stress responses [6].

Once considered “evolutionary junk,” non-coding RNAs (ncRNAs) are now known to regulate numerous molecular mechanisms and exert a profound influence on the development and organization of living organisms [7]. Plant regulatory non-coding RNAs can be broadly classified into two main groups based on length [8]: small non-coding RNAs [sncRNAs; 18–30 nucleotides (nt)] and long non-coding RNAs (lncRNAs; >200 nt). Small non-coding RNAs include microRNAs (miRNAs), small interfering RNAs (siRNAs), phased secondary siRNAs (phasiRNAs), and related classes. In addition, the non-coding RNA collection in living cells also includes circular non-coding RNAs (circRNAs) [8,9]. Although lncRNAs have been identified across a wide range of eukaryotic genomes, including several plant species such as *Arabidopsis thaliana* [8], *Zea mays* [9], *Oryza sativa* [10], *Jatropha curcas* [11], and *Brassica napus* [12], they exhibit relatively low sequence conservation at the nucleotide level. lncRNAs act as regulators of tissue and cell-specific plant gene expression through a combination of transcriptional, post-transcriptional, and epigenetic modifications [13]. In plants, lncRNAs are known to be expressed in response to environmental stimuli such as drought [14], salinity [15,16], heat [17], light [18], nutrients [9,10], pathogen response [12,19], plant immunity and growth [20]. In addition, several studies indicated crucial roles played by lncRNAs in growth and development, including seed development [21,22], seed ageing [23], seed dormancy [24], seed germination [15], seed vigor [25], plant reproduction [26], and senescence [27,28].

Maize (*Zea mays*) is one of the most significant and widely cultivated crops globally. Its versatility and extensive use make it essential for global food security, agriculture, and economies. One of the major threats to maize storage and consumption is the attack of seed-colonizing fungus (*Aspergillus flavus*) and the resultant production of aflatoxin in the kernels. *A. flavus* is a ubiquitous filamentous fungus that occurs as both a soil-dwelling saprophyte and an opportunistic pathogen of plants, animals, and humans, with the highest prevalence in warm tropical and subtropical regions. The species exhibits high genetic and phenotypic diversity, enabling survival across multiple ecological niches and complicating long-term aflatoxin management [29,30]. Aflatoxin contamination in maize carries critical implications for international trade and public health [29], as they are class I carcinogens and pose severe risks to both human and animal health, leading to conditions such as hepatotoxicity, teratogenicity, immunotoxicity, and acute aflatoxicosis, especially in developing countries. Aflatoxin contamination could lead to significant financial losses for the maize industry, with estimates ranging from US$52.1 million to US$1.68 billion annually in the United States [30]. These losses are primarily due to reduced crop yields, increased production costs, and the need for testing and regulatory compliance to ensure safe aflatoxin levels in maize products.

Extensive studies on the genetic regulation of aflatoxin biosynthesis, along with its associated physiological, biochemical, and molecular aspects, have been reported by several researchers [31,32,33,34,35,36,37]. These studies detailed the regulation of aflatoxin biosynthesis by nutritional and environmental conditions and highlighted the significant impact of both biotic and abiotic factors on aflatoxin accumulation. Investigations into aflatoxin resistance in maize have led to the identification of quantitative trait loci (QTLs), genes, and proteins associated with resistance to aflatoxin accumulation [38,39,40,41,42,43]. RNA-binding proteins (RBPs) function as central post-transcriptional regulators that control RNA splicing, transport, stability, and translation, thereby shaping cellular responses to biotic stress [44]. In the maize–*A. flavus* crosstalk, resistance is a complex quantitative trait involving coordinated expression of numerous defense-related genes and regulatory networks, including RNA-mediated pathways [45]. Emerging studies indicate that small and long non-coding RNAs participate in antifungal responses by modulating transcription factors, kinases, and stress-response genes, highlighting the importance of RNA-level regulation during infection [42,46]. Within this regulatory layer, lncRNAs can interact with RBPs as molecular scaffolds or decoys, influencing the stability and translation of defense-related mRNAs and thereby controlling the accumulation of pathogenesis-related proteins and other resistance factors [47]. The action of elongation factor-Tu (EF-Tu), a widely known RBP in the induction of lncRNA, *At5NC056820* in *Arabidopsis* [48], and the interaction of plant argonaute protein with non-coding RNAs like lncRNA [49] are recognized as crucial for cellular responses under stress conditions [50]. Moreover, microRNA profiling of maize accessions revealed the importance of zma-miR156-squamosa promoter binding protein (SBP) and zma-miR398/zma-miR394-F-box combinations as driving the resistance mechanisms through interaction with transcription factors and protein kinases [42]. The noncoding RNA interactome, especially the lncRNA–RBP–mRNA interactions are likely to fine-tune maize immune responses, including oxidative stress signaling, pathogen recognition, and systemic defense activation, which are critical for limiting *A. flavus* colonization and aflatoxin accumulation [51]. The relevance of lncRNAs expressed in *A. flavus* to maize host resistance remains unclear, despite the availability of such reports for major fungal pathogens affecting other crops [12,19,20]. Moreover, significant differential expression of lncRNAs in *A. flavus* under stress conditions, including water deficit, elevated CO_2_, and temperature fluctuations, was reported [52]. Building on this foundation, the present study focuses on identifying differentially expressed lncRNAs with critical roles in the maize-*A. flavus* crosstalk, as well as elucidating the molecular interactions between these lncRNAs and receptor proteins, thereby advancing our understanding of maize resistance to aflatoxin contamination. Moreover, the study aims to explore the structure-function relationships of lncRNA and protein interactions and their effect on transcriptional regulation and biological processes in maize. This will be helpful in the identification of lncRNA-mediated regulatory mechanisms that can be harnessed for developing aflatoxin-resistant varieties.

## 2. Results

### 2.1. Differential Expression of lncRNAs Under A. flavus Infection

**Figure 1:**
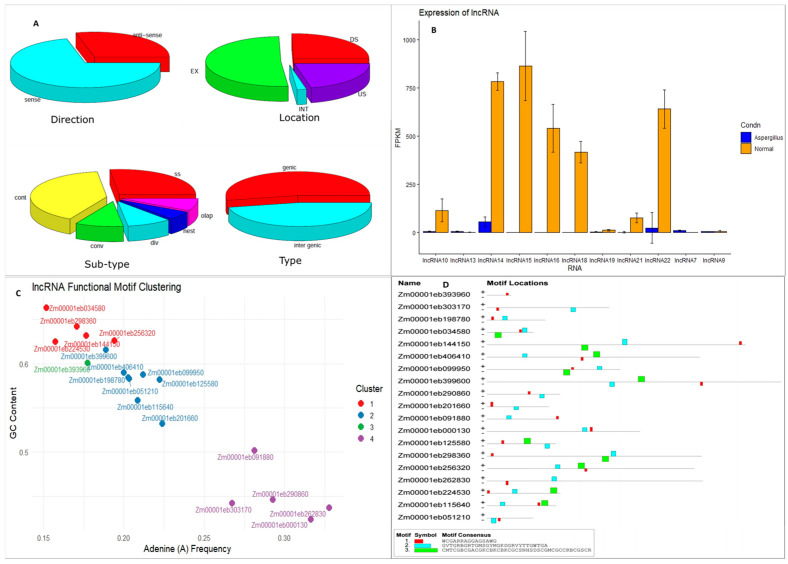
The characterization of differentially expressed lncRNAs under normal germination and *A. flavus* infection conditions in maize (**A**) classification of lncRNAs based on direction, location, and type. The direction of lncRNA occurrence in the genome is presented as sense and anti-sense, whereas the location of occurrence is given as exonic (EX), intronic (INT), upstream (US), and downstream (DS). The sub-type classification refers to convergent (conv), divergent (div), containing (cont), same strand (ss), overlapping (olap), and nested (nest). The types of lncRNAs represented include genic and intergenic. (**B**) Expression profile during *A. flavus* infection and normal germination. The FPKM values are given for differentially expressed lncRNAs, while SD is depicted as error bars; orange for normal germination and blue for *A. flavus* infection (**C**). Clustering of lncRNAs based on A and GC content (**D**). The top three MEME motifs identified and their distribution in the lncRNAs studied.

Stress-responsive lncRNAs act as molecular switches fine-tuning *Zea mays* expression to biotic and abiotic stresses and are identified by transcriptome dataset analysis. A total of 2104 lncRNAs were discovered in maize by analyzing the datasets SRR13414919 (*A. flavus* inoculated) and SRR19536700 (normal germination). Based on direction, 614 of them were antisense RNAs, while 1490 were sense RNAs. Likewise, 53% of the lncRNAs were of genic origin, while 47% were intergenic. Based on genomic location, 51% were located in the exonic regions, whereas the remaining lncRNAs were distributed across downstream, upstream, and intronic regions. The classification of lncRNAs is given in detail in Figure 1A. Among these 461 lncRNAs were found to be expressed differentially under *A. flavus* infection. *Zm00001eb144150* and *Zm00001eb406410* were recorded to have higher expressions under normal germination, whereas *Zm00001eb303170* was found to have higher expression under *A. flavus* infection. We selected the top 21 differentially expressed lncRNAs for further analysis. Although FlExible Extraction of LncRNAs (FEElnc) classified *Zm00001eb124380* as an lncRNA, further analysis revealed it as a protein-coding gene and hence was not considered in the analysis. The lncRNAs showing high levels of expression under normal as well as *A. flavus* infection are given in Figure 1B. The lncRNAs 1, 7, 14, and 21 were found to be expressed under *A. flavus* infection, wherein lncRNA1 (*Zm00001eb303170*) was found to have higher expression under fungal infection. Under normal germination, lncRNA5 and lncRNA6 were found to have higher expression. The lncRNA sequences are listed as Supplementary Information (SI-1).

### 2.2. Classification and Clustering of lncRNAs Based on Sequence Features

Classification of lncRNAs based on sequence features is necessary to gain insights into the potential roles played by lncRNAs in gene regulation, protein binding, and functional conservation across species. The differentially expressed lncRNA sequences were analyzed for GC content as well as mono and dinucleotide frequencies. The results were subjected to an unsupervised machine learning algorithm (K-means clustering) for identifying potential functional motifs. Based on Adenine (A) and GC content, the lncRNAs were grouped into 4 clusters (Figure 1C). Cluster 2 included eight lncRNAs, while clusters 1 and 4 had 5 members each. Cluster 3 contained only a single lncRNA, *Zm00001eb393960* (lncRNA2). GC content was higher in the first cluster, while the frequency of ‘A’ was higher for cluster 4. The meme analysis discovered three motifs in the lncRNA sequences, of which motif 2 was associated with GO terms (GO: 0009507, GO: 0009570, and GO: 0009535) associated with chloroplast stroma and chloroplast thylakoid membranes. This motif was present in all the lncRNA sequences except lncRNA2 (Figure 1D). Motif 3 was associated with GO terms (GO: 0009507, GO: 0009570, GO: 0005739, GO: 0000 166, and GO: 0080008). This motif is associated with molecular function, nucleotide binding, and cellular components, namely mitochondria, chloroplast stroma, and the CUL4 RING ubiquitin ligase complex. This motif was present in K-clusters 1 and 2.

### 2.3. Secondary Structure of lncRNAs

**Figure 2:**
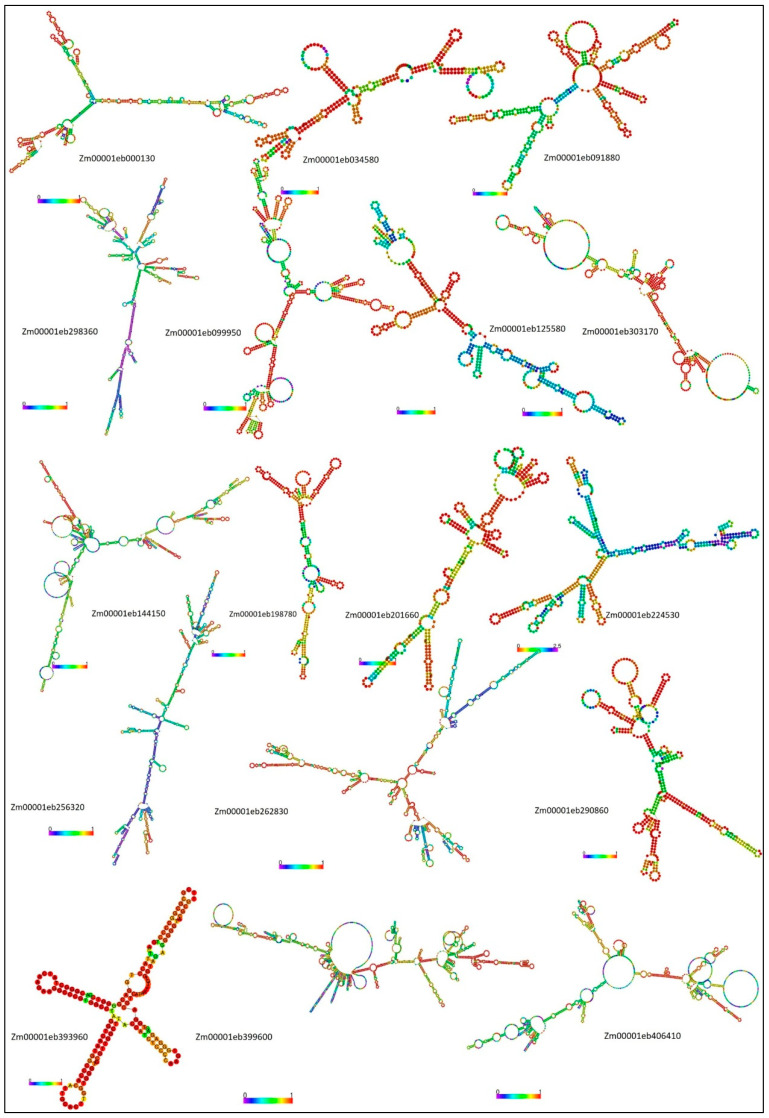
The predicted secondary structures of differentially expressed lncRNAs in maize during *A. flavus* infection and normal germination are represented, indicating the in silico folding. The stable stem loop structure offers binding sites for receptor proteins like RBP, indicating a possible role in post-transcriptional regulation during the maize–*A. flavus* interaction. The color variation from blue to red represents the pairing confidence score from 0 to 1.

The secondary structures of lncRNAs play a crucial role in molecular recognition by receptor proteins, in maintaining the structural stability of the lncRNA, and in identifying their functions in transcriptional regulation. The predicted secondary structures of lncRNAs with roles in maize seed germination and *A. flavus* response using the RNAfold web server are depicted in Figure 2. The structures of individual lncRNAs varied from simple, stable structures with long stems and hairpin loops and high pairing confidence (mostly red), as in *Zm00001eb393960*, to highly branched, containing multiple long-range interactions and large unpaired loops, which are indicators of flexible and dynamic function as in *Zm00001eb224530* and *Zm00001eb406410*. The RNA secondary structure prediction output details are given in Table 1. The free energy of the thermodynamic ensemble of the RNA structure varied from −75.81 to −768.36 kcal/mol. The highest (negative) energy values were recorded for *Zm00001eb144150* and *Zm00001eb399600*. The high negative value of free energy is an indication of extremely high thermodynamic stability of the RNA molecules and the ability of the lncRNAs to form complex, well-structured scaffolds—possibly involved in regulatory or structural functions. The frequency of the Minimum Free Energy (MFE) structure in the ensemble appears quite low (0–3.25) and is an indication of the structural flexibility of lncRNAs. This conformational heterogeneity, allowing the RNA to exist in several alternate forms with similar free energies, is common for large flexible RNAs, including lncRNA, indicating their roles in regulatory function and protein interactions. The greater structural variations and adoption of many diverse confirmations are also reflected in the high ensemble diversity, ranging from 12.1 to 526.89.

For lncRNAs with predicted tertiary structures, stereochemical validation metrics (ERRAT scores and Ramachandran plots) were employed as supportive indicators of model quality. The structural validation of lncRNAs revealed a very high ERRAT score (>95%) represented as the Overall Quality Factor (OQF) for lncRNAs 2, 3, 4, 8, and 22. The Ramachandran plots indicated that more than 90% of residues were found in the favored regions in the structures of lncRNAs 4, 8, 13, 17, and 22. Most of the lncRNAs, except lncRNAs 5, 19, and 21, have no residues in the disallowed regions, which is an indication of stable protein structure with good stereochemical quality and structural accuracy, making it a crucial tool for validating protein models. However, lncRNAs 6 and 9 failed to have any protein homology structure. Although ERRAT scores and Ramachandran plots are traditionally used for protein structure validation, they were applied in this study as proxy stereochemical assessments of predicted lncRNA tertiary models generated using template-based or ab initio approach. These metrics were used cautiously to evaluate backbone geometry, residue torsion consistency, and gross structural anomalies rather than protein-specific folding accuracy. The Ramachandran plots of lncRNAs are given as SI-2.

The lncRNA sequences were probed for the presence of binding domains of heterogeneous nuclear ribonucleoprotein (hnRNP) proteins, which are essential for their regulatory roles. The lncRNAs contained multiple binding sites for PTBP1 (Polypyrimidine Tract Binding Protein 1) and hnRNP K domains. Structural homology also revealed the existence of domains similar to eukaryotic ELAVL1/HuR (Human antigen R), TIA1 (T-cell intracellular antigen 1), and LIN28 among the maize lncRNAs. However, none of the lncRNAs contained binding sites for the PUM (Pumilio proteins) domain. The details are given in Table 2.

### 2.4. RNA-Binding Proteins in lncRNA Regulation

Long non-coding RNAs (lncRNAs) play diverse regulatory roles during maize kernel germination under both normal and *A. flavus*-colonizing conditions, modulating gene expression at the transcriptional and translational levels. Interactions between lncRNAs and RNA-binding proteins (RBPs) are central to post-transcriptional regulation of mRNA and the control of protein synthesis. Because protein synthesis is a fundamental process governing seed germination and stress responses, the present study investigated the interactions between differentially expressed maize lncRNAs and major RBPs selected as receptor proteins. RNA-binding proteins, including branch point bridging protein (*Zm00001eb421010*), KH domain protein (*Zm00001eb102180*), transcription factor for RNA polymerase TFIID (*Zm00001eb320850*), elongation factor (*Zm00001eb432840*) containing RRM domain, DNA-directed RNA polymerase (*Zm00001eb408310*), pentatricopeptide repeat-containing protein (*Zm00001eb108470*), and argonaute 2 (*Zm00001eb070760*), which is an important component of RISC (RNA-Induced Silencing Complex). The functions of the receptor proteins are given as SI-3, and protein structures of receptors are given as SI (zip file).

### 2.5. Molecular Docking Analysis of lncRNA-RBP Interactions

**Figure 3:**
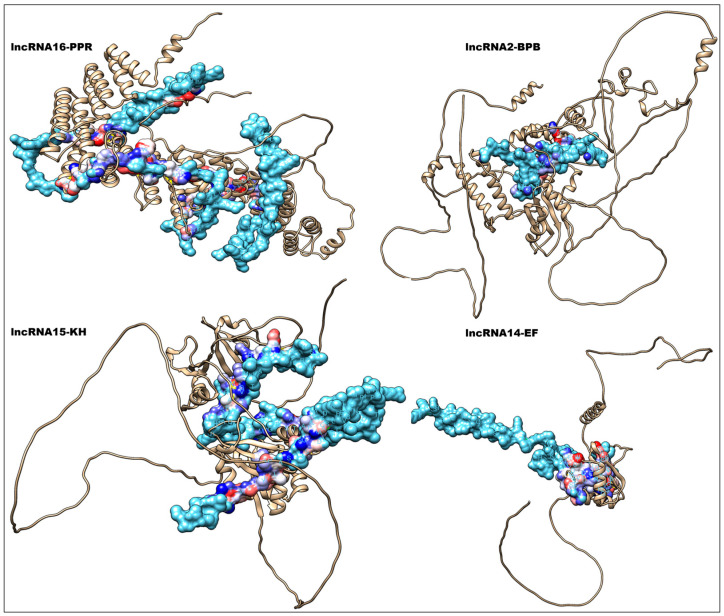
Predicted three-dimensional model of the lncRNA–RNA-binding protein (RBP) interactions in maize and *A. flavus* crosstalk. The lncRNA backbone is shown as a ribbon, while the RBP is displayed as a helical structure with surface representation at the interaction interface. Surface colors indicate electrostatic potential, where red represents negatively charged regions, blue indicates positively charged regions, and white denotes neutral areas. The contrasting charge distribution highlights the potential binding interface between the lncRNA and the RBP, suggesting electrostatic complementarity that may stabilize the complex during post-transcriptional regulation. The interaction models are given for RBPs such as branch point bridging protein (BPB), K-homology domain protein (KH), elongation factor containing RRM domain, and pentatricopeptide repeat-containing protein (PPR).

**Figure 4:**
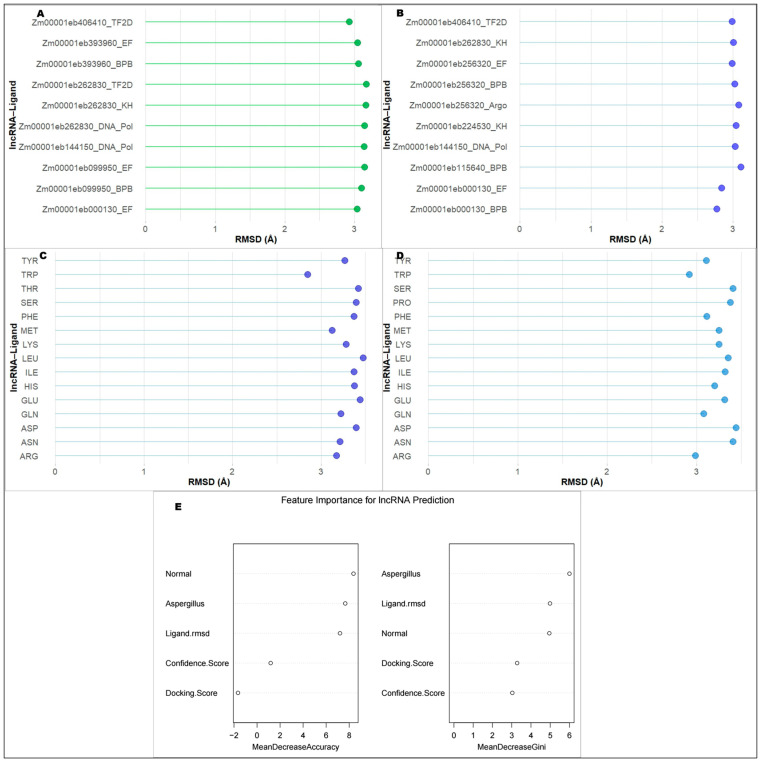
Prediction of lncRNA–RBP interactions using machine learning algorithms to identify the optimal lncRNA–RBP combinations based on (**A**) ligand surface and (**B**) receptor surface docking parameters, determined by the lowest RMSD values and interaction counts. The favorable amino acid residues are represented as (**C**) interacting residues on the ligand surface and (**D**) interacting residues on the receptor surface. The feature importance plot of the random forest model developed by combining the gene expression data under normal germination and *A. flavus* inoculation, along with the receptor protein-ligand docking, including features like docking free energy, binding probability, and ligand RMSD, is given in (**E**). Colour dots denote ligand and receptor surface.

Molecular docking serves as a crucial in silico, structure-based technique to study interactions between lncRNAs and RBPs, providing a rapid alternative to predict binding poses, affinities, and specific interaction sites. Among the docked complexes, the highest binding energy was observed for lncRNA1 (*Zm00001eb303170*) and branch point bridging (BPB) protein: −375.37 kcal/mol. This complex also recorded the highest binding probability or confidence score of 0.9891, while the ligand RMSD (Root Mean Square Deviation) values were high (40.85 A^0^). High binding energies (>300 kcal/mol) and high confidence scores (>0.96) were noticed for docked complexes viz; lncRNA4-KH, lncRNA2-EF, lncRNA15-KH, lncRNA4-EF and lncRNA15-TF2D. The lowest RMSD value observed was 10.73 and 12.37 A^0^, respectively, for the docking complexes lncRNA16-PPR and lncRNA14-EF. The details are given in Table 3. Similarly, the complex lncRNA15-KH also recorded a low RMSD value of 15.11 A^0^. The details of important lncRNA-receptor protein docking complexes are given in Figure 3. We used ML algorithms to classify the interacting residues from the receptor as well as the ligand interfaces. From the receptor interface, TRP was identified as the most favorable amino acid based on the mean RMSD values. Other favorable residues include ARG, GLN, TYR, and PHE. Tryptophan was the most favorable amino acid on the ligand interface, whereas the other favorable residues include MET, ARG, ASN, and GLN. The most favorable lncRNA-protein binding complexes based on the least RMSD values based on receptor surface are lncRNA13-BPB, lncRNA13-EF, and lncRNA16-EF, whereas from the ligand surface, they are lncRNA6-TF2D, lncRNA13-EF, and lncRNA2-EF. All the details are given in Figure 4A–D.

A random forest model was created by converging gene expression data under normal germination and *A. flavus* inoculation, along with the receptor protein-ligand docking, including docking free energy, binding probability, and ligand RMSD. The variable importance plot indicated that binding probability and docking score or free energy are the most important variables, followed by gene expression under normal germination. The details are given in Figure 4E. Based on this model, the best lncRNA-receptor protein combinations were identified for normal germination as well as under *A. flavus* infection based on the highest confidence and the lowest docking score. These are lncRNA17-KH and lncRNA1-BP, respectively, for normal germination and under *A. flavus* infection.

### 2.6. Interactions of Maize lncRNAs with Selected RBPs

When TFIID was used as the receptor, lncRNA15 exhibited the lowest docking free energy (−308.87 kcal mol^−1^) and the highest binding probability (0.96), whereas lncRNA6 showed the lowest RMSD. Both lncRNA15 and lncRNA6 are highly expressed under normal germination of maize kernels. The receptor interface analysis revealed that PHE, ARG, TYR, ILE, and LYS were the most favorable amino acids, while on the ligand interface, MET, TRP, GLN, HIS, and ASN were the favorable amino acids. The best lncRNA interacting with TFIID was lncRNA6, based on both receptor and ligand interfaces, with the least RMSD values. Details are in SI.

The analysis revealed that the docking free energy and binding probabilities were higher for the binding of lncRNA1 with the branch point bridging protein (BPB). The observed values were −375.35 kcal/mol and 0.9891. The lowest RMSD values were noticed for the binding of *Zm00001eb393960* (lncRNA2) with BPB. The expression of both lncRNA1 and lncRNA2 was higher under *A. flavus* infection conditions. The ML algorithms identified GLN, LYS, HIS, TRP, and MET as the favorable amino acids on the receptor interface, while HIS, TRP, ISE, GLN, and ARG were on the ligand interface. Based on low RMSD values, lncRNA13 and lncRNA16 have better binding with BPB on the receptor interface, while on the ligand interface, lncRNA2 and lncRNA7 were better. Details are in SI-5.

The highest binding probability (0.9795) and lowest docking free energy (−343.26 kcal/mol) were exhibited by the binding of *Zm00001eb034580* (lncRNA4) with the KH domain protein. But for this complex, the ligand RMSD values were high, 25.62 A^0^. However, the binding of lncRNA15 (*Zm00001eb298360*) with KH recorded the lowest RMSD value of 15.11 A^0^, together with low binding energy and high confidence score of −327.47 kcal/mol and 0.9721, respectively. The most favorable amino acids on the receptor interface were ARG, TYR, PHE, GLN and LYS while the lowest RMSD mean score for KH binding on the receptor interface was for lncRNA17. On the ligand interface, TRP, MET, GLN, TYR and PHE had the lowest RMSD value and the stable docking pose was by lncRNA17 and lncRNA4. The KH protein was effective in binding with lncRNAs expressed both under normal germination and *A. flavus* infection conditions. Details are in SI-6.

When the elongation factor was used as the receptor protein, the binding energies of the docking complexes ranged from −249.89 to −328.53 kcal/mol. Similarly, the binding probabilities were also high, with a range of 0.8806 to 0.9726. The lowest RMSD values were noticed for the binding of lncRNA1 (18.98 A^0^), lncRNA2 (17.83 A^0^), lncRNA7 (16.19 A^0^), and lncRNA14 (12.37 A^0^). EF was also effective in binding with lncRNAs under *A. flavus* infection, as well as normal germination. The favorable amino acids on the receptor interface are TRP, ARG, PHE, MET, and TYR. On the ligand interface, TRP, ASP, LYS, HIS, and PHE were found to be favorable in achieving stability in the docking complex. Based on mean RMSD values across the receptor and ligand interface, lncRNA13 was marked as the ideal binding partner. Details are in SI-7.

The highest binding probability (0.9449) and lowest docking free energy (−292.09 kcal/mol) were exhibited by the binding of *Zm00001eb224530* (lncRNA18) with DNA-directed RNA polymerase. However, the mean RMSD values were quite high for this complex (46.58 A^0^). Additionally, the binding of *Zm00001eb303170* (lncRNA1) as ligand resulted in high binding probability, low free energy, and low RMSD values of −268.8 kcal/mol, 0.915, and 19.28 A^0^, respectively. The favorable amino acids on the receptor interface are TRP, HIS, MET, GLN, and TYR, and on the ligand surface, they are TRP, ARG, TYR, MET, and ASN. lncRNA5 (*Zm00001eb144150*) was identified as the best binding partner with DNA-directed RNA polymerase based on low RMSD values. Details are in SI-8.

The docking free energy and binding probabilities were higher for the binding of lncRNA2 with argonaute 2 protein, while the lowest RMSD values were noticed for the binding of lncRNA16. Based on RMSD values, the favorable amino acids on the receptor surface were TRP, GLN, ARG, ILE, and GLU, while on the ligand surface, TRP, ARG, TYR, GLU, and ASN were favorable. Based on the receptor surface residue analysis, lncRNA16 and lncRNA19 exhibited better binding with argonaute protein, while lncRNA2 and lncRNA20 were favorable from the ligand surface analysis. Details are in SI-9.

Pentatricopeptide repeat (PPR) proteins of mitochondria play key roles in post-transcriptional regulation and RNA metabolism. The docking free energy and binding probabilities were higher for the binding of lncRNA1 with PPR protein, while the lowest RMSD values were noticed for the binding of lncRNA16. The favorable amino acids on the receptor interface are TRP, GLN, ASN, MET, and ARG, and on the ligand surface are PHE, MET, HIS, TRP, and TYR. lncRNA13 and lncRNA14 were identified as the best binding partners with PPR proteins based on ML algorithm results. A strong induction of lncRNA14 was noticed upon normal germination compared to *A. flavus* infection. Details are in SI-10.

### 2.7. lncRNA-miRNA-mRNA Interaction Under Normal Germination and A. flavus Infection

**Figure 5:**
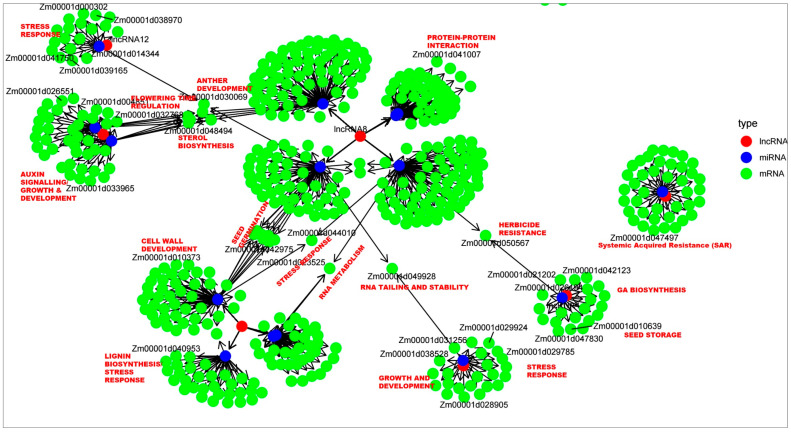
Integrative lncRNA–miRNA–protein competing endogenous RNA (ceRNA) regulatory network associated with maize defense against *A. flavus* infection and aflatoxin contamination. The network comprises 9 differentially expressed lncRNAs and their interacting 22 miRNAs, showing regulatory connections with corresponding maize target proteins. In the network, lncRNAs act as miRNA sponges, preventing suppression of defense genes. The network nodes depict lncRNAs, miRNAs, and genes, with edges showing predicted regulatory interactions from expression data and computational predictions.

Non-coding RNA (ncRNA) target prediction using the psRNATarget server enables the identification of potential interactions between ncRNAs (such as lncRNAs or miRNAs) and their target transcripts, primarily in plant systems. By inputting the differentially expressed lncRNA sequences as either query (small RNA) or targets, we identified the underlying miRNAs. A total of 22 miRNAs were identified from 9 lncRNAs submitted as targets. Details are in Table 4. Further, we used these miRNAs as a query to identify the protein targets. This data was used to draw an lncRNA-miRNA-mRNA regulatory network. Based on degree centrality, lncRNA8 and lncRNA16, as well as the miRNAs, zma-miR164a-3p, zma-miR160c-3p, zma-miR399d-5p, and zma-miR399d-5p were found to have maximum interactions. The targeted maize proteins based on maximum interaction include *Zm00001d014753* (pre-mRNA-splicing factor SYF2), *Zm00001d023525* (pentatricopeptide repeat-containing protein), *Zm00001d032768* (CCT motif family protein), and *Zm00001d048494* (hydroxymethylglutaryl-CoA synthase). Details are given in Figure 5 and SI-11. The lncRNA-miRNA-protein interaction network details the major functions as stress response, seed germination, specialized metabolite synthesis, systemic acquired resistance (SAR), growth and development, hormone signaling, RNA metabolism, RNA stability, and seed storage. From the listed target candidates, we selected candidates with expectation values less than 2.5 and tried docking with the lncRNA containing the miRNA involved in the inhibition activity. The targets identified by the psRNAtarget server include laccase, GRAS transcription factor, aquaporin, pollen-specific kinase partner protein, adaptin ear-binding coat-associated protein 2, calmodulin, etc. The details are in SI-12. This tool is particularly useful for exploring regulatory roles of lncRNAs, including their function as miRNA precursors, decoys, or direct regulators of mRNA transcripts. The predictions are based on customizable parameters such as expectation scores and unpaired energy values, offering insights into the post-transcriptional regulatory networks mediated by ncRNAs.

## 3. Discussion

A plant resistance strategy based on the inherent ability to survive, recover, and reproduce under adverse environmental conditions is a complex trait involving the interplay of multiple genes at molecular, metabolic, and physiological levels. The adaptation to adverse environmental conditions consists of the expression of a series of functional proteins and regulatory elements, of which the long non-coding RNAs are a major part [53]. Differential expression of non-coding RNAs, including miRNAs and lncRNAs, under adverse environmental conditions has been documented in several plant species, such as *Arabidopsis thaliana* [8,13,54], *Oryza sativa* [10,55], *Solanum lycopersicum* [56], *Zea mays* [20,53,57], *Triticum aestivum* [17], and *Arachis hypogaea* [23]. The studies reported the regulatory roles of lncRNA in different stress conditions, such as heat, drought, salinity, nutrient, disease resistance, as well as in several physiological processes like germination, growth, leaf senescence, seed development, seed aging, dormancy, and spikelet fertility. Like the protein-coding genes, lncRNAs also exhibit differential expression during stress conditions in addition to the tissue and development-specific functions [45,51,58]. In the present study, we observed the differential expression of lncRNAs under normal and *Aspergillus flavus* infection conditions in maize, especially the unique expressions of lncRNA 1 under fungal infection and lncRNA5 and 6 under normal germination conditions. Moreover, the strong induction of lncRNA14 (>14 fold) under normal germination compared to the low level of expression under *A. flavus* infection indicates the high relevance in maize growth and development. These differentially expressed lncRNAs can serve as biomarkers for *A. flavus* resistance and can act as breeding targets for improving maize yield, quality, and disease resistance [59].

The interaction of lncRNAs with DNA, RNA, and proteins plays a crucial role in regulating several cellular and molecular processes underlying the aforementioned conditions. Moreover, lncRNA molecules also regulate mRNA stability, splicing, and degradation processes in addition to binding with RBPs [47,60]. The interaction between lncRNAs and RNA-binding proteins is currently understood as a plausible mechanism underlying lncRNA functions. The transcriptional regulation activity of lncRNAs is mostly by binding to DNA-binding proteins like transcriptional factors and also to RNA polymerase II [61] through recruiting the regulatory protein complex, inhibition, or decoy, or by the transcription of the lncRNA [47]. The biological regulator role of lncRNA in plants [62] is achieved through regulation of gene expression at the transcription, post-transcription, and epigenetic level, targeting various stress-responsive mRNAs, regulatory gene(s) encoding transcription factors, and numerous microRNAs (miRNAs). The modulation of TF activity through binding of lncRNAs has been documented in plants like rice and *Arabidopsis,* wherein the molecular mechanism is listed as promoter region interaction, modification of chromatin structure, or through induction of epigenetic modifications [47,62,63]. The role of lncRNA1840 in tomato fruit maturation involves binding with several RNA-binding proteins, including the elongation factor [64], with a similar EF-lncRNA interaction reported in *Arabidopsis* [48]. Alternative splicing events, which increase the transcriptomic and proteomic complexities in living organisms, are known to be controlled by lncRNAs through the interaction with RNA-binding proteins, including splicing factors, leading to the development of several diseases in humans [47,60], while it is associated with stress response in plants [51,65]. The interaction of lncRNAs with RBPs and specialized metabolites leading to enhanced resistance to fungal and viral pathogens has been reported in crops like tomato, rice, and *Gossypium hirsutum* [51,66].

In the present study, the target protein (receptors) included various RNA-binding proteins like the branch point bridging protein, the K homology domain-containing protein, transcription factor TFIID, elongation factor, DNA-directed RNA polymerase, and argonaute 2 protein [48,49,50,51]. Among these, Splicing Factor1 (SF1) or BPB is required for splicing of pre-mRNA and functions in the recognition of the branch site (5′-UACUAAC-3′), the pyrimidine tract, and the 3′-splice site at the 3′-end of introns. An additional RRM motif existed in plants compared to the animal and yeast homologs, indicating a potential role in conservation in the plant lineage [15]. The multiple binding sites found for PTBP1 in the studies of lncRNAs are an indication of their role in mRNA splicing. Similarly, the KH domain is a well-studied RNA-binding domain with roles in pre-mRNA splicing as well as stress signaling and DNA damage response [67]. The essential components of transcription, like the basal transcription factor TFIID, are crucial for RNA polymerase II (pol II) promoter recognition and transcription initiation and elongation factors for regulating transcription elongation. The studied lncRNAs exhibited strong interactions with the transcription machinery, thereby affecting the functionality of nearby genes. Argonaute-2 proteins perform important biological functions of regulatory ncRNA-mediated gene silencing as well as miRNA generation [49,68]. Similarly, PPR proteins participate in diverse post-transcriptional processes within plant mitochondria and plastids, where they are essential for regulating plant growth, development, fertility restoration in cytoplasmic male sterility systems, and adaptation to both biotic and abiotic stresses mostly through RNA processing, maturation, and editing [69]. The results highlight that the studied lncRNAs are not isolated transcriptional byproducts but active regulatory molecules embedded in a complex interaction network involving splicing, transcription, stress response, and RNA silencing machinery. Their ability to associate with multiple RBPs and transcriptional regulators suggests a multifunctional regulatory potential, enabling plants to fine-tune gene expression programs in response to developmental cues and environmental challenges.

Understanding structure–function relationships has been fundamental for deciphering biological processes at the molecular level. Since the rise of structural biology, three-dimensional protein structures have provided key insights into protein–protein interactions, ligand recognition, complex assembly, and self-organization, which are central to both basic biology and medical research [50,70]. lncRNAs display extensive functional versatility, largely due to their ability to associate with diverse protein partners through multiple molecular mechanisms [50]. Such protein interactions are essential for lncRNAs to perform their regulatory roles. Structural characterization of lncRNA–protein complexes, therefore, offers a strong framework for understanding their molecular functions and may ultimately support the design of new strategies to manage biotic and abiotic stress in plants [50,51]. lncRNAs regulate gene expression by partnering with proteins involved in processes such as DNA repair, chromatin remodeling, and transcriptional control. Their secondary structures—for example, hairpins formed by intramolecular base pairing—play an important role in determining RBP accessibility by either exposing or masking binding regions. Conversely, RBPs can reshape RNA structure upon interaction. Importantly, lncRNAs often undergo structural changes in response to environmental conditions, highlighting their dynamic nature and regulatory capacity [50,71]. The confirmational heterogeneity of lncRNAs with similar free energy regimes was crucial for their flexibility in regulatory roles and protein binding in the present study, also.

lncRNAs are involved in the regulation of hormone signaling pathways through interactions with miRNA and mRNA [42,46,56]. Through complementary sequences known as miRNA response elements (MREs), miRNAs can interact with target mRNAs, leading to mRNA decay or translational repression of the target mRNAs. The miRNA abundance is controlled not only during transcription and precursor processing but also through interactions with other RNA molecules that can bind and inhibit them via partial sequence complementarity. The lncRNAs, acting as decoys of specific miRNAs known as endogenous target mimics (eTMs) or competing endogenous RNAs (ceRNAs), sequester miRNAs by mimicking their target sites, thereby reducing the ability of miRNAs to repress their natural gene targets [72]. miRNAs and their target genes are involved in maize response to biotic and abiotic stresses, notably *A. flavus* infection [42,46]. This study reports that miRNAs are the regulators of aflatoxin resistance in maize, especially zma-miR156–squamosa promoter binding protein (SBP) and zma-miR398/zma-miR394–F-box combinations, playing vital roles in plant response. In the present study, lncRNA22 is found to harbor zma-miR156e-3p, which is involved in systemic acquired resistance against *A. flavus,* involving the maize candidate gene ‘flavin monooxygenase’ [42]. The interaction of maize miRNA ‘Zma-miRNA319’ with MYB transcription factor ZmMYB74 in maize imparts stalk rot resistance in maize due to modification in lignin deposition [46]. Moreover, 9 differentially expressed lncRNAs were found to have MREs and interacted with 22 different miRNAs. Among them, zma-miR171k-5p, zma-miR171h-5p, zma-miR162-5p, zma-miR164c-3p, zma-miR164h-3p, zma-miR399d-5p, zma-miR160c-3p, zma-miR171h-3p, and zma-miR171k-3p were already reported as miRNA decoys or targets in maize [73]. The miR396 family has been well characterized in *Sorghum bicolor*, and GRF (Growth Regulating Factor) genes were identified as the target [74]. The resistance to *A. flavus* in groundnut also depended on miR396 and miR-156e-3p [75]. The transcriptional regulation of lncRNAs involves the interaction with transcriptional factors like WRKY and MYB, leading to stress responses [3]. The docking study results, as well as the miRNA regulatory landscape, indicate that lncRNA and protein interactions are crucial to decipher stress signaling as well as growth and development in maize. The lncRNA-RBP interactions are crucial for understanding the roles played by lncRNAs in deeper biological processes, as well as the structure-function relationship of lncRNAs. This will help devise novel strategies for stress resistance and aflatoxin contamination in maize, including targeted interventions like gene editing, for seed, grain, and human health, while reducing the economic damage [51].

The role of lncRNAs in plant defense is well documented, particularly through their co-expression with functional genes [76]. This mechanism is largely associated with the reduction in reactive oxygen species (ROS) accumulation and the regulation of plant defense genes and transcription factors [3,46,77,78]. Interactions between lncRNAs and RNA-binding proteins (RBPs) containing the RNA recognition motif (RRM) domain have been identified as key factors in tumor progression and antiviral responses [79,80]. In plants, lncRNA–RBP interactions have been reported [81], with more than 5000 lncRNAs identified across species such as *A. thaliana*, *A. lyrata*, *Populus trichocarpa*, and *Z.mays*. In the present study, differentially expressed maize lncRNAs were found to interact with key RNA-binding and transcription-related proteins during *A. flavus* infection. These interactions modulate transcription initiation, RNA processing, translation, and RNA-silencing pathways, thereby fine-tuning defense gene expression and stress adaptation. As a result, they contribute to enhanced host resistance and reduced aflatoxin contamination. Aflatoxin contamination in maize poses a severe challenge to the food, feed, and seed industries due to its impact on food safety, trade, and economic sustainability [29,30]. Understanding the roles of lncRNAs in maize–*A. flavus* interactions provide novel molecular insights that can be directly utilized for crop improvement [40]. From an industry viewpoint, this research is highly significant as it offers new biomarkers and regulatory targets for developing aflatoxin-resistant maize varieties through precision breeding and biotechnological approaches. Such advancements can reduce post-harvest losses, minimize dependence on costly fungicides, and ensure compliance with stringent international food safety standards, thereby enhancing global market competitiveness [82]. By integrating cutting-edge functional genomics with applied crop protection, this study addresses a critical industry need and supports long-term strategies for food security and economic growth.

## 4. Materials and Methods

### 4.1. Data Sources and Retrieval

#### 4.1.1. Genomic Data

The genome, transcriptome, and proteome sequences of *Zea mays* inbred line B73 were retrieved from EnsemblPlants (https://plants.ensembl.org/Zea_mays/Info/Index (accessed on Thursday, 7 March 2024)).

#### 4.1.2. Gene Expression Data

RNA-seq datasets generated on the Illumina platform were obtained from the NCBI Sequence Read Archive (SRA) under the accession PRJNA691427, corresponding to the study “Comparative transcriptome profiling uncovers the key genes linked to early-stage resistance to *A. flavus* in maize” by Liu et al. [83]. In this experiment, mature ears from resistant (AF99) and susceptible (AF32) maize lines were inoculated with *A. flavus* spores. Kernels collected 15 days after pollination were longitudinally divided; one-half was treated with a spore suspension for 5 min, while the other was immersed in sterile water and used as the 0 h control. A minimum of four kernel halves were pooled per sample at five time points: 0 h (T0), 0.5 h (T1), 1.5 h (T2), 3 h (T3), and 6 h (T4). For this study, we selected the following datasets: SRR13414919, SRR13414925, SRR13414940 (6 h); SRR13414922, SRR13414943, SRR13414944 (3 h); SRR13414921, SRR13414923, SRR13414947 (1.5 h); SRR13414934, SRR13414935, SRR13414936 (0.5 h); and SRR13414937, SRR13414938, SRR13414939 (0 h). Additionally, RNA-seq data from germinating maize seeds of the B73 variety (PRJNA842522) were included for comparative analysis between normal germination and *A. flavus*-infected kernels. The experiments used for this comparison were SRR19536700, SRR19536701, and SRR19536702. A general protocol followed for inoculation of *A. flavus* in maize kernels is provided in Supplementary Information (SI-13).

### 4.2. Identification of lncRNAs in Maize and Their Differential Expression

The analysis of SRA data was conducted utilizing the Galaxy online workflow [84]. The data, downloaded in FASTQ format, underwent quality assessment and adapter trimming through Trimmomatic version 0.38.1. High-quality reads were aligned using Hisat2 version 2.2.2 (http://ccb.jhu.edu/software/hisat2/index.shtml (accessed on Thursday, 7 March 2024)) [85] and subsequently assembled with Cufflinks, which facilitated the estimation of their abundances across various RNA-Seq samples, expressed as FPKM values [86]. The differential gene expression analysis was performed comparing Normal versus *A. flavus* inoculated samples (combined), as well as Normal versus *A. flavus* inoculations separately at time points of 0, 0.5, 1.5, 3.0, and 6.0 h, in addition to comparing different stages of *A. flavus* inoculation (0, 0.5, 1.5, and 3.0 h versus 6.0 h). The fold change values were calculated based on the FPKM values of *A. flavus* infection and normal germination. Differentially expressed lncRNAs were identified using an adjusted *p*-value (FDR) cutoff of <0.05 and a minimum |log_2_FC| ≥ 1. All genes presented showed highly significant adjusted *p*-values and strong fold changes, confirming robust differential regulation under *A. flavus* infection. This analysis was executed using the Cuffdiff package version 2.2.1.6. The assembled transcripts from Cufflinks and the genome sequence in FASTA format were utilized for the prediction of lncRNAs using the tool FEElnc (FlExible Extraction of Long non-coding RNA) version 0.2.1 and further annotated [87].

### 4.3. Secondary and Tertiary Structures of lncRNA

The minimum free energy secondary structures of lncRNAs were predicted using the RNAfold web server (http://rna.tbi.univie.ac.at/cgi-bin/RNAWebSuite/RNAfold.cgi accessed on Tuesday 10 September 2024) from the FASTA sequences of lncRNAs. The differentially expressed lncRNA sequences were translated using the Expasy (https://web.expasy.org/translate/ accessed on Tuesday 10 September 2024) tool, and these protein sequences were utilized for homology modeling of tertiary structure using SWISS-MODEL (https://swissmodel.expasy.org/ (accessed on Tuesday 10 September 2024)). The best model was saved in PDB format for further analyses. The PDB structures of lncRNAs were evaluated with the SAVESv6.1 server accessed at https://saves.mbi.ucla.edu/ (accessed from Thursday 21 August 2025). We used the programs ERRAT [88] and PROCHECK [89] to validate the lncRNA structures. The selected differentially expressed lncRNA sequences were subjected to sequence similarity in the RNAcentral web server (https://rnacentral.org/ accessed from Wednesday, 11 September 2024) to identify similar sequences.

### 4.4. Receptor Proteins and Structure

RNA-binding proteins (RBPs) interacting with the selected lncRNAs were first predicted using LncPro (http://cmbi.bjmu.edu.cn/lncpro (This website is presently not accessible, the last access was on Friday, 27 September 2024)). Only RBP families showing statistically significant interaction scores with the lncRNAs were retained. From these families, maize homologs were identified using gene annotation databases, namely EnsemblPlants (https://plants.ensembl.org/Zea_mays/Info/Index (accessed from Thursday, 7 March 2024)) and Phytozome (https://phytozome-next.jgi.doe.gov/info/Zmays_Zm_B73_REFERENCE_NAM_5_0_55 (accessed on Monday, 11 March 2024)). To ensure biological relevance under the experimental conditions, candidate RBPs were further filtered based on their expression levels in the analyzed SRA datasets, and only those exhibiting high and consistent expression were selected as receptor proteins for molecular docking analyses. From the identified RNA-binding protein families, we selected the maize homologs that had high expression in the analyzed SRA accessions as the target receptor for molecular docking simulation studies. The protein sequences of the target receptors were used for homology-based protein modeling using SWISS-MODEL (https://swissmodel.expasy.org// (accessed on Monday, 30 September 2024)). The resultant PDB files were used for molecular docking studies. The RBP motifs in lncRNA sequences were identified with the R package Biostrings using a custom R code.

### 4.5. Molecular Docking Simulation Studies

The RNA-binding proteins were considered as receptors and lncRNAs as ligands, with the PDB files rendered as an input to the HDOCK server (http://hdock.phys.hust.edu.cn/ accessed on Thursday, 10 October 2024) to simulate docking. The best docking model was selected based on minimum RMSD values and high binding affinities. This docking model in PDB format was used as input for the UCSF ChimeraX tool to identify the interacting amino acids. The best model identified using HDOCK was used for machine learning analyses for ligand-protein interaction analyses.

### 4.6. lncRNA–miRNA–mRNA Interactions

Non-coding RNA (ncRNA) target prediction using the psRNATarget server (https://www.zhaolab.org/psRNATarget/ (accessed on Thursday, 17 July 2025)) was utilized for the identification of miRNA target sequences present in the lncRNA sequences. The lncRNA sequences were utilized as targets initially, and the underlying miRNAs were identified. Subsequently, these miRNAs were used as a query to identify the target proteins. The interaction lncRNA-miRNA-mRNA interaction information was used to develop the interaction network using R packages igraph, ggraph, and tidyverse. The network was further analyzed based on parameters, betweenness centrality, and degree centrality in descending order.

### 4.7. Machine Learning Analyses

Using the best HDOCK models for each of the lncRNA–receptor protein interactions, a cumulative interaction file was created for each RBP. This contained information on each of the interacting residues on the ligand as well as the receptor side and the RMSD values, and was used for machine learning analyses to identify the top interacting residues, as well as the best lncRNA interacting with each of the RBPs. The dataset was split 60:40 into training and testing sets for machine learning analyses and validated via 5-fold cross-validation. Model accuracy was assessed using confusion matrices. All the analyses were done in R-studio (version 4.1.1) using packages dplyr, ggplot2, xgboost, Matrix, and forcats. Additionally, a random forest model was developed by including gene expression data under normal germination and *A. flavus* inoculation conditions, along with the receptor protein-lncRNA docking attributes, including docking free energy, binding probability, and ligand RMSD, using random forest and caret packages in R-studio.

## 5. Conclusions

This study demonstrates that long noncoding RNAs (lncRNAs) play central roles in *Zea mays* defense responses against *Aspergillus flavus* infection as well as in normal developmental processes like seed germination. The lncRNAs were found to exhibit distinct expression patterns under fungal infection and normal germination conditions, especially lncRNA1 (unique induction during fungal infection) and lncRNA14 (high induction during germination), highlighting their potential as molecular markers for growth, development, and pathogen resistance. Moreover, maize lncRNAs interact with a wide array of RNA-binding proteins (RBPs), including splicing factors, transcription factors, elongation factors, argonaute proteins, and pentatricopeptide repeat proteins. These interactions are crucial for regulating RNA processing, transcription, and RNA silencing pathways, thereby positioning lncRNAs as active components of stress adaptation and gene regulation networks rather than passive transcriptional byproducts. Several stress-related miRNAs, including zma-miR156, zma-miR398, zma-miR394, and zma-miR396, were found to be associated with differentially expressed lncRNAs, reinforcing their roles in systemic acquired resistance and aflatoxin resistance. The differentially expressed lncRNAs identified in the study can serve as promising biomarkers for resistance breeding and as molecular targets for improving maize yield, quality, and resilience against fungal pathogens. Above all, this study is highly relevant to the maize industry as it identifies lncRNA-mediated regulatory mechanisms that can be harnessed for developing aflatoxin-resistant varieties. By reducing contamination risks, it supports food safety compliance, minimizes economic losses, and enhances global trade competitiveness.

## Figures and Tables

**Table 1:** Predicted RNA secondary structure features of maize lncRNAs, including free energy of the thermodynamic ensemble, frequency of the minimum free energy (MFE) structure, and ensemble diversity. Chromosomal locations and RNAcentral identifiers are also provided. Tertiary structure quality assessments were performed on predicted RNA 3D models using stereochemical validation tools, including ERRAT overall quality factor (OQF) and favorable residues (Fav_res) from Ramachandran plot statistics, applied here as proxy indicators of backbone geometry and structural consistency. lncRNAs lacking suitable template-based 3D models are indicated as NA.

lncRNA	Maize_Gene_ID	Free Energy of the Thermodynamic Ensemble (kcal/mol)	Frequency of the MFE Structure in the Ensemble (%)	Ensemble Diversity	RNAcentral_ID	Location	OQF	Fav_res (%)
lncRNA1	*Zm00001eb303170*	−173.68	3.25	12.1	zm00001e033098	Chr07	91.6667	83.7
lncRNA2	*Zm00001eb393960*	−75.81	0	122.32	zm00001e038121	Chr09	100	73.5
lncRNA3	*Zm00001eb198780*	−150.71	0	65.69	zm00001e024281	Chr04	96	88
lncRNA4	*Zm00001eb034580*	−133.42	0.05	41.34	zm00001e003498	Chr01	96.2963	98.9
lncRNA5	*Zm00001eb144150*	−768.36	0	526.89	zm00001e018913	Chr03	68.9655	67.6
lncRNA6	*Zm00001eb406410*	−535.77	0	413.9	zm00001e039342	Chr10	NA	NA
lncRNA7	*Zm00001eb099950*	−359.77	0	154	zm00001e009864	Chr02	86.6667	77.3
lncRNA8	*Zm00001eb399600*	−763.85	0	482.16	zm00001e038667	Chr09	97.619	93.4
lncRNA9	*Zm00001eb290860*	−139.95	0	72.33	zm00001e031946	Chr06	NA	NA
lncRNA11	*Zm00001eb201660*	−143.38	0	66.25	zm00001e024578	Chr04	75	83.3
lncRNA12	*Zm00001eb091880*	−136.87	0	86.12	zm00001e009062	Chr02	80	77.8
lncRNA13	*Zm00001eb000130*	−243.02	0	195.93	zm00001e000013	Chr01	94.8718	97.9
lncRNA14	*Zm00001eb125580*	−154.1	0	118.15	zm00001e017041	Chr03	90.1961	82.9
lncRNA15	*Zm00001eb298360*	−650.18	0	520.63	zm00001e032669	Chr07	82.2222	84.8
lncRNA16	*Zm00001eb256320*	−591.69	0	508.23	zm00001e015987	Chr05	8.69565	53.2
lncRNA17	*Zm00001eb262830*	−404.03	0	364.8	zm00001e029338	Chr06	93.1373	91.8
lncRNA18	*Zm00001eb224530*	−206.8	0	144.64	zm00001e012955	Chr05	87.5	80.8
lncRNA19	*Zm00001eb115640*	−139.84	0	135.19	zm00001e011343	Chr02	94.5205	72.4
lncRNA20	*Zm00001eb051210*	−97.64	0.05	38.38	zm00001e106964	Chr01	89.2857	81.6
lncRNA21	*Zm00001eb206550*	−100.1	0.18	43.99	zm00001e114877	Chr04	0	43.9
lncRNA22	*Zm00001eb371660*	−189.75	0	124.43	zm00001e036039	Chr09	97.4684	91.9

**Table 2:** Predicted Heterogeneous Nuclear Ribonucleoprotein (hnRNP) binding motifs identified within differentially expressed maize lncRNAs during *A. flavus* infection. The table shows the presence of specific protein recognition sequences and their corresponding start positions within each lncRNA. The binding sites probed include PTBP1 (Polypyrimidine Tract Binding Protein 1), hnRNP K domain, ELAVL1/HuR (Human antigen R), TIA1 (T-cell intracellular antigen 1), LIN28, and PUM (Pumilio proteins) domain. These motifs indicate potential interactions between lncRNAs and RNA-binding proteins involved in transcriptional and post-transcriptional regulation.

lnCRNA	HuR	PTBP1	TIA1	FUS	HNRNPK	LIN28	PUM1
	AUUUA	UCUUC	UUUU	GGUG	UCCC	GGAG	UGUA[ACU]AUA
*Zm00001eb393960*	NA	NA	NA	71, 75	94	104	NA
*Zm00001eb303170*	504	135, 186, 390, 498	46, 98, 423, 564, 751, 788	276, 742	49, 101, 149, 265, 366	NA	NA
*Zm00001eb198780*	1	369	109, 137	116, 194	NA	197	NA
*Zm00001eb034580*	NA	NA	NA	88, 94, 160, 232	168	237, 249, 259	NA
*Zm00001eb144150*	1655	NA	244	25, 433, 529, 741, 792, 844, 880, 1132, 1174, 1356, 1362, 1371	21, 428, 562, 999, 1181, 1329, 1400, 1427	853, 1042, 1188, 1204, 1263, 1491, 1582, 1604, 1643, 1666	NA
*Zm00001eb406410*	NA	42, 619, 679	NA	192, 218, 233, 242, 257, 308, 434, 455, 518, 671, 873, 887, 984, 991, 1113	48, 54, 87, 381, 639, 769, 847, 1211, 1310	147, 239, 251, 801, 943, 1127	NA
*Zm00001eb099950*	676, 848	NA	844	61, 412, 446, 593, 717	565	41, 415, 638, 831, 854	NA
*Zm00001eb399600*	NA	206, 546, 684, 882, 1404, 1467	1592, 1668, 1770	367, 460, 508, 562, 670, 919, 982, 1039, 1066, 1138, 1156, 1165, 1372, 1475, 1656	431, 537, 608, 689, 701, 720, 927, 1706, 1744	14, 439, 565, 763, 769, 862, 1042, 1051, 1075, 1141, 1273, 1444	NA
*Zm00001eb290860*	NA	213, 355	NA	NA	248, 400	137, 205	NA
*Zm00001eb201660*	90	336	116	252	NA	33	NA
*Zm00001eb091880*	NA	328	135	NA	338	301, 313, 408	NA
*Zm00001eb000130*	62	577	174,567, 607, 888	50, 908	18	116, 181, 438, 444, 459, 677, 767	NA
*Zm00001eb125580*	NA	418	NA	132, 209, 329, 359	293, 344	116	NA
*Zm00001eb298360*	NA	NA	NA	124, 396, 414, 738, 839, 957, 1315, 1385, 1390	364, 730	275, 566, 659, 684, 773, 1130, 1158, 1360, 1370	NA
*Zm00001eb256320*	NA	181, 355, 862	1304	98, 227, 422, 560, 659, 695, 1259, 1288, 1308	94, 370, 394, 865, 1331	110, 119, 128, 146, 230, 266, 281, 302, 344, 428, 443, 461, 500, 689, 749, 896, 980	NA
*Zm00001eb262830*	1340	94, 121, 952	1216, 1366, 1392	423, 518, 655, 1059, 1270	100, 209, 235, 970	565, 1297, 1352	NA
*Zm00001eb224530*	NA	224, 233, 416	NA	129, 159, 168, 179, 318	NA	11, 243, 374, 461	NA
*Zm00001eb115640*	NA	247	7	172	37, 99, 183, 250, 289	178	NA
*Zm00001eb051210*	NA	NA	NA	160	146	NA	NA
*Zm00001eb206550*	NA	163	NA	11, 281	169	86, 217, 243	NA
*Zm00001eb371660*	NA	156	409, 533	100	32, 446	125, 204, 235, 240	NA

NA—Absence of hnRNP binding sites.

**Table 3:** Predicted interactions between maize lncRNAs and RNA-binding proteins (RBPs) based on molecular docking analysis. The RBPs were treated as receptor proteins and the lncRNAs as ligands, and the interactions were evaluated using docking scores, confidence scores, and ligand RMSD values. More negative docking scores indicate stronger binding affinity, higher confidence scores reflect greater reliability of the predicted complex, and lower RMSD values suggest more stable and accurate binding conformations.

Sl. No	LncRNA	*Receptor*	Ligand	Docking Score	Confidence Score	Ligand RMSD (Å)
1	lncRNA1	*Zm00001eb303170*	TF2D	−252.47	0.8859	31.33
2	lncRNA2	*Zm00001eb393960*	TF2D	−275.26	0.9245	29.13
3	lncRNA14	*Zm00001eb125580*	TF2D	−221.76	0.8077	33.83
4	lncRNA4	*Zm00001eb034580*	TF2D	−269.1	0.9154	29.23
5	lncRNA5	*Zm00001eb144150*	TF2D	−217.65	0.7946	69.71
6	lncRNA10	*Zm00001eb124380*	TF2D	−201.98	0.7388	39.99
7	lncRNA6	*Zm00001eb406410*	TF2D	−215.41	0.7872	25.24
8	lncRNA12	*Zm00001eb091880*	TF2D	−236.18	0.8486	50.86
9	lncRNA16	*Zm00001eb256320*	TF2D	−239.08	0.8559	49.24
10	lncRNA13	*Zm00001eb000130*	TF2D	−192.99	0.7026	43.21
11	lncRNA15	*Zm00001eb298360*	TF2D	−308.87	0.96	41.54
12	lncRNA11	*Zm00001eb201660*	TF2D	−278.14	0.9284	47.44
13	lncRNA17	*Zm00001eb262830*	TF2D	−230.72	0.834	39.7
14	lncRNA18	*Zm00001eb224530*	TF2D	−214.46	0.784	40.92
15	lncRNA19	*Zm00001eb115640*	TF2D	−201.23	0.7359	39.1
16	lncRNA20	*Zm00001eb051210*	TF2D	−222.54	0.8101	37.81
17	lncRNA1	*Zm00001eb303170*	BP	−375.37	0.9891	40.85
18	lncRNA2	*Zm00001eb393960*	BP	−243.17	0.8657	27.93
19	lncRNA10	*Zm00001eb124380*	BP	−198.25	0.7241	23.06
20	lncRNA19	*Zm00001eb115640*	BP	−233.62	0.8419	38.92
21	lncRNA7	*Zm00001eb099950*	BP	−210.69	0.771	41.49
22	lncRNA14	*Zm00001eb125580*	BP	−226.84	0.823	48
23	lncRNA15	*Zm00001eb298360*	BP	−251.02	0.8829	50.09
24	lncRNA4	*Zm00001eb034580*	BP	−261.91	0.9036	49.8
25	lncRNA5	*Zm00001eb144150*	BP	−230.68	0.8339	57.83
26	lncRNA11	*Zm00001eb201660*	BP	−217.85	0.7953	34.09
27	lncRNA12	*Zm00001eb091880*	BP	−218.94	0.7988	56.5
28	lncRNA16	*Zm00001eb256320*	BP	−234.58	0.8444	40.62
29	lncRNA13	*Zm00001eb000130*	BP	−208.42	0.7629	44.24
30	lncRNA17	*Zm00001eb262830*	BP	−246.18	0.8725	39.28
31	lncRNA18	*Zm00001eb224530*	BP	−210.84	0.7715	39.47
32	lncRNA20	*Zm00001eb051210*	BP	−243.86	0.8673	33.53
33	lncRNA3	*Zm00001eb198780*	BP	−221.32	0.8063	23.73
34	lncRNA2	*Zm00001eb393960*	KH	−272.53	0.9206	17.44
35	lncRNA4	*Zm00001eb034580*	KH	−343.26	0.9795	25.62
36	lncRNA7	*Zm00001eb099950*	KH	−261.37	0.9027	25.81
37	lncRNA10	*Zm00001eb124380*	KH	−221.53	0.807	3.37
38	lncRNA11	*Zm00001eb201660*	KH	−263.72	0.9067	36.07
39	lncRNA14	*Zm00001eb125580*	KH	−239.47	0.8569	22.2
40	lncRNA15	*Zm00001eb298360*	KH	−327.47	0.9721	15.11
41	lncRNA16	*Zm00001eb256320*	KH	−272.98	0.9213	21.16
42	lncRNA19	*Zm00001eb115640*	KH	−211.48	0.7737	23.8
43	lncRNA20	*Zm00001eb051210*	KH	−270.61	0.9178	22.97
44	lncRNA1	*Zm00001eb303170*	KH	−297.71	0.9505	37.45
45	lncRNA13	*Zm00001eb000130*	KH	−233.34	0.8411	41.92
46	lncRNA18	*Zm00001eb224530*	KH	−235.53	0.8469	49.05
47	lncRNA17	*Zm00001eb262830*	KH	−289.47	0.9421	31.49
48	lncRNA12	*Zm00001eb091880*	KH	−236.52	0.8495	40.29
49	lncRNA5	*Zm00001eb144150*	KH	−268.89	0.9151	69.85
50	lncRNA3	*Zm00001eb198780*	KH	−237.78	0.8527	30.73
51	lncRNA10	*Zm00001eb124380*	EF	−203.99	0.7465	20.13
52	lncRNA2	*Zm00001eb393960*	EF	−328.53	0.9726	17.83
53	lncRNA15	*Zm00001eb298360*	EF	−284.35	0.9363	31.59
54	lncRNA19	*Zm00001eb115640*	EF	−240.32	0.8589	29.59
55	lncRNA20	*Zm00001eb051210*	EF	−242.66	0.8645	22.1
56	lncRNA7	*Zm00001eb099950*	EF	−224.73	0.8168	16.19
57	lncRNA14	*Zm00001eb125580*	EF	−220.01	0.8022	12.37
58	lncRNA1	*Zm00001eb303170*	EF	−301.03	0.9535	18.98
59	lncRNA4	*Zm00001eb034580*	EF	−321.23	0.9685	22.89
60	lncRNA5	*Zm00001eb144150*	EF	−232.05	0.8377	58.56
61	lncRNA11	*Zm00001eb201660*	EF	−287.62	0.94	26.86
62	lncRNA12	*Zm00001eb091880*	EF	−205.03	0.7504	32.85
63	lncRNA13	*Zm00001eb000130*	EF	−240.62	0.8597	23.37
64	lncRNA16	*Zm00001eb256320*	EF	−234.62	0.8445	20.69
65	lncRNA18	*Zm00001eb224530*	EF	−232.29	0.8383	23.25
66	lncRNA17	*Zm00001eb262830*	EF	−249.89	0.8806	29.46
67	lncRNA3	*Zm00001eb198780*	EF	−255.39	0.8917	25.93
68	lncRNA2	*Zm00001eb393960*	Argo	−282.45	0.9339	37.28
69	lncRNA7	*Zm00001eb099950*	Argo	−239.13	0.856	32.33
70	lncRNA14	*Zm00001eb125580*	Argo	−224.96	0.8175	50.25
71	lncRNA1	*Zm00001eb303170*	Argo	−264.19	0.9075	53.23
72	lncRNA4	*Zm00001eb034580*	Argo	−265.03	0.9089	51.3
73	lncRNA5	*Zm00001eb144150*	Argo	−245.72	0.8715	74.05
74	lncRNA10	*Zm00001eb124380*	Argo	−237.98	0.8532	41.8
75	lncRNA16	*Zm00001eb256320*	Argo	−246.72	0.8737	29.57
76	lncRNA12	*Zm00001eb091880*	Argo	−223.24	0.8123	47.81
77	lncRNA11	*Zm00001eb201660*	Argo	−246.85	0.874	59.53
78	lncRNA15	*Zm00001eb298360*	Argo	−258.2	0.897	31.36
79	lncRNA13	*Zm00001eb000130*	Argo	−196.02	0.7151	45.88
80	lncRNA17	*Zm00001eb262830*	Argo	−240.74	0.8599	49.8
81	lncRNA18	*Zm00001eb224530*	Argo	−221.04	0.8055	48.24
82	lncRNA19	*Zm00001eb115640*	Argo	−203.66	0.7452	59.23
83	lncRNA20	*Zm00001eb051210*	Argo	−230.77	0.8342	36.32
84	lncRNA19	*Zm00001eb115640*	DNA_pol	−211.83	0.775	38.97
85	lncRNA14	*Zm00001eb125580*	DNA_pol	−234.85	0.8451	44.17
86	lncRNA1	*Zm00001eb303170*	DNA_pol	−268.8	0.915	19.28
87	lncRNA2	*Zm00001eb393960*	DNA_pol	−242.83	0.8649	20.43
88	lncRNA4	*Zm00001eb034580*	DNA_pol	−278.22	0.9285	37.67
89	lncRNA18	*Zm00001eb224530*	DNA_pol	−292.09	0.9449	46.58
90	lncRNA5	*Zm00001eb144150*	DNA_pol	−216.66	0.7914	63.99
91	lncRNA16	*Zm00001eb256320*	DNA_pol	−213.41	0.7804	41.17
92	lncRNA15	*Zm00001eb298360*	DNA_pol	−265.28	0.9093	29.32
93	lncRNA13	*Zm00001eb000130*	DNA_pol	−207.99	0.7613	30.18
94	lncRNA12	*Zm00001eb091880*	DNA_pol	−222.33	0.8095	35.56
95	lncRNA7	*Zm00001eb099950*	DNA_pol	−208.8	0.7642	40.2
96	lncRNA11	*Zm00001eb201660*	DNA_pol	−232.58	0.8391	24.49
97	lncRNA10	*Zm00001eb124380*	DNA_pol	−205.7	0.7529	17.61
98	lncRNA17	*Zm00001eb262830*	DNA_pol	−243.24	0.8659	36.69
99	lncRNA20	*Zm00001eb051210*	DNA_pol	−225.45	0.8189	35.62
100	lncRNA3	*Zm00001eb198780*	DNA_pol	−265.92	0.9104	27.84
101	lncRNA2	*Zm00001eb393960*	Pathogenesis-related protein 10	−201.47	0.7368	27.86
102	lncRNA7	*Zm00001eb099950*	Pathogenesis-related protein 10	−185.91	0.6722	24.59
103	lncRNA16	*Zm00001eb256320*	Pentatricopeptide	−237.32	0.8515	10.73
104	lncRNA20	*Zm00001eb051210*	Pentatricopeptide	−242.33	0.8637	32.06
105	lncRNA2	*Zm00001eb393960*	Pentatricopeptide	−284.26	0.9361	27.07
106	lncRNA7	*Zm00001eb099950*	Pentatricopeptide	−222.14	0.8089	22.17
107	lncRNA14	*Zm00001eb125580*	Pentatricopeptide	−217.51	0.7942	48.5
108	lncRNA15	*Zm00001eb298360*	Pentatricopeptide	−256.47	0.8937	36.68
109	lncRNA5	*Zm00001eb144150*	Pentatricopeptide	−233.65	0.842	51.68
110	lncRNA1	*Zm00001eb303170*	Pentatricopeptide	−308.23	0.9595	39.96
111	lncRNA11	*Zm00001eb201660*	Pentatricopeptide	−277.49	0.9276	29.13
112	lncRNA12	*Zm00001eb091880*	Pentatricopeptide	−221.2	0.8060	23.91
113	lncRNA13	*Zm00001eb000130*	Pentatricopeptide	−212.75	0.7782	28
114	lncRNA17	*Zm00001eb262830*	Pentatricopeptide	−242.43	0.864	34.21
115	lncRNA18	*Zm00001eb224530*	Pentatricopeptide	−246.94	0.8742	41.92
116	lncRNA19	*Zm00001eb115640*	Pentatricopeptide	−232.14	0.8379	25.31
117	lncRNA3	*Zm00001eb198780*	Pentatricopeptide	−240.18	0.8586	31.17

**Table 4:** Predicted lncRNA–miRNA–mRNA interaction network in maize under normal germination and *A. flavus* infection. The table lists the involved lncRNAs, interacting miRNAs, their target maize mRNAs, and the mode of inhibition. Cleavage indicates miRNA-mediated degradation of the target transcript, suggesting a regulatory role of lncRNAs in modulating gene expression during stress and normal conditions.

lncRNA	Maize Target Gene	miRNA_Acc.	Target_Acc.	Inhibition
lncRNA11	*Zm00001eb201660*	zma-miR171h-5p	Zm00001eb201660_T001	Cleavage
lncRNA11	*Zm00001eb201660*	zma-miR171k-5p	Zm00001eb201660_T001	Cleavage
lncRNA11	*Zm00001eb201660*	zma-miR396e-3p	Zm00001eb201660_T001	Cleavage
lncRNA4	*Zm00001eb034580*	zma-miR162-5p	Zm00001eb034580_T001	Cleavage
lncRNA16	*Zm00001eb256320*	zma-miR2275b-5p	Zm00001eb256320_T001	Cleavage
lncRNA16	*Zm00001eb256320*	zma-miR390a-3p	Zm00001eb256320_T001	Cleavage
lncRNA16	*Zm00001eb256320*	zma-miR390b-3p	Zm00001eb256320_T001	Cleavage
lncRNA16	*Zm00001eb256320*	zma-miR399a-5p	Zm00001eb256320_T001	Cleavage
lncRNA12	*Zm00001eb091880*	zma-miR167b-3p	Zm00001eb091880_T001	Cleavage
lncRNA17	*Zm00001eb262830*	zma-miR164c-3p	Zm00001eb262830_T004	Cleavage
lncRNA17	*Zm00001eb262830*	zma-miR164h-3p	Zm00001eb262830_T004	Cleavage
lncRNA17	*Zm00001eb262830*	zma-miR164d-3p	Zm00001eb262830_T004	Cleavage
lncRNA6	*Zm00001eb406410*	zma-miR171d-5p	Zm00001eb406410_T001	Cleavage
lncRNA6	*Zm00001eb406410*	zma-miR171e-5p	Zm00001eb406410_T001	Cleavage
lncRNA19	*Zm00001eb115640*	zma-miR397a-5p	Zm00001eb115640_T001	Translation
lncRNA19	*Zm00001eb115640*	zma-miR397b-5p	Zm00001eb115640_T001	Translation
lncRNA8	*Zm00001eb399600*	zma-miR399d-5p	Zm00001eb399600_T001	Cleavage
lncRNA8	*Zm00001eb399600*	zma-miR160c-3p	Zm00001eb399600_T001	Cleavage
lncRNA8	*Zm00001eb399600*	zma-miR164a-3p	Zm00001eb399600_T001	Translation
lncRNA8	*Zm00001eb399600*	zma-miR164c-3p	Zm00001eb399600_T001	Cleavage
lncRNA8	*Zm00001eb399600*	zma-miR164h-3p	Zm00001eb399600_T001	Cleavage
lncRNA8	*Zm00001eb399600*	zma-miR171h-3p	Zm00001eb399600_T001	Translation
lncRNA8	*Zm00001eb399600*	zma-miR171k-3p	Zm00001eb399600_T001	Translation
lncRNA22	*Zm00001eb371660*	zma-miR156e-3p	Zm00001eb371660_T001	Cleavage

## Data Availability

The original contributions presented in this study are included in the article/Supplementary Materials. Further inquiries can be directed to the corresponding author. The original data links are given as URLs in the article. All the other relevant information regarding PDB structures, lncRNA sequences, and docking complexes is deposited in the corresponding author’s Mendeley account https://data.mendeley.com/drafts/bd29dvx2j5.

## Supplementary Materials

The following supporting information can be downloaded at: https://www.mdpi.com/article/10.3390/ijms27052493/s1.

## Abbreviations

lncRNA	Long non-coding RNA
RBP	RNA-binding protein
miRNA	Micro RNA
FPKM	Fragments Per Kilobase of transcript per Million mapped reads
BPB	Branch point bridging protein
KH	KH domain protein
TFIID	Transcription factor for RNA polymerase TFIID
EF	Elongation factor containing the RRM domain
PPR	Pentatricopeptide repeat-containing protein
