# Supplementary material for: Integrative Analysis of lncRNA–RBP (RNA-Binding Protein) Regulatory Networks Reveals Molecular Targets for Enhancing Zea mays Resistance to Aspergillus flavus and Aflatoxin Contamination
Source: Int J Mol Sci. 2026 Mar 8;27(5):2493. doi: 10.3390/ijms27052493 (PMC12986324; doi:10.3390/ijms27052493)
Supplement: Supplementary file 1 [file ijms-27-02493-s001.zip › SI-13.pdf]

Supplementary Information (SI-13)

Protocol followed for *Aspergillus* inoculation

Fungal strain and inoculum preparation

The fungal cultures (*Aspergillus flavus* strains NAIMCC-F-01918 and NAIMCC-F-00204) used for inoculation were procured from the ICAR–National Bureau of Agriculturally Important Microorganisms (NBAIM), Mau, India. These isolates are well documented for their high aflatoxin-producing potential and were therefore selected for experimental use.

Inoculum was prepared as a conidial suspension. The fungus was cultured on Potato Dextrose Agar (PDA) medium in Petri dishes and incubated at 28 °C for 12–14 days. Conidia were harvested by gently washing the agar surface with sterile distilled water containing 0.1% Tween-20 (two drops per 100 mL). The suspension was filtered through four layers of sterile cheesecloth to remove mycelial debris. Conidial concentration was determined using a hemocytometer and adjusted to 1×10^8 conidia mL^{-1} with sterile distilled water. All inoculum required for a given experimental year was prepared at a single time point and stored at 4 °C as an aqueous suspension. On the day of inoculation, the stock suspension was diluted to 2.0×10^7 conidia mL^{-1} and maintained on ice until use.

Maize experimentation

Healthy maize kernels harvested from local landraces (IMR-130, IMR-185, IMR-431, and IMR-487) maintained at the ICAR-NISST Regional Station, Bengaluru, were used for experimentation. Seeds were surface-sterilized using 1.0% sodium hypochlorite prior to treatment. The experiment included inoculation with *A. flavus* strains NAIMCC-F-01918 and NAIMCC-F-00204, with two replications per strain. A control treatment without fungal inoculum (hydro-primed seeds) was also included. Seeds were soaked in the prepared fungal inoculum or sterile distilled water (control) for three hours before further analysis.
