# Supplementary material for: Integrative Analysis of lncRNA–RBP (RNA-Binding Protein) Regulatory Networks Reveals Molecular Targets for Enhancing Zea mays Resistance to Aspergillus flavus and Aflatoxin Contamination
Source: Int J Mol Sci. 2026 Mar 8;27(5):2493. doi: 10.3390/ijms27052493 (PMC12986324; doi:10.3390/ijms27052493)
Supplement: Supplementary file 1 [file ijms-27-02493-s001.zip › SI-2.pdf]

SI-2 The Ramachandran plots of lncRNAs

lncRNA1

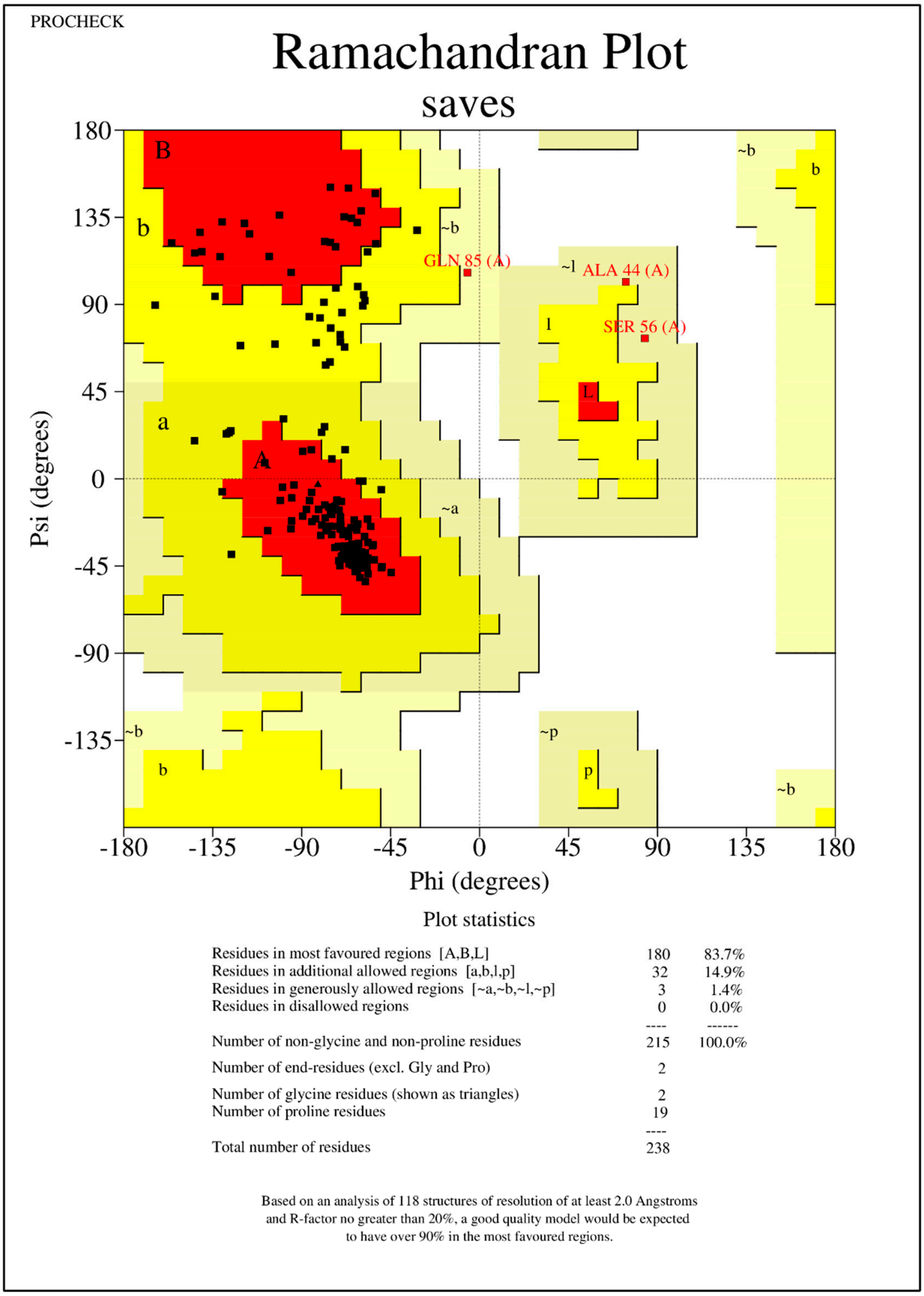

PROCHECK

Ramachandran Plot

saves

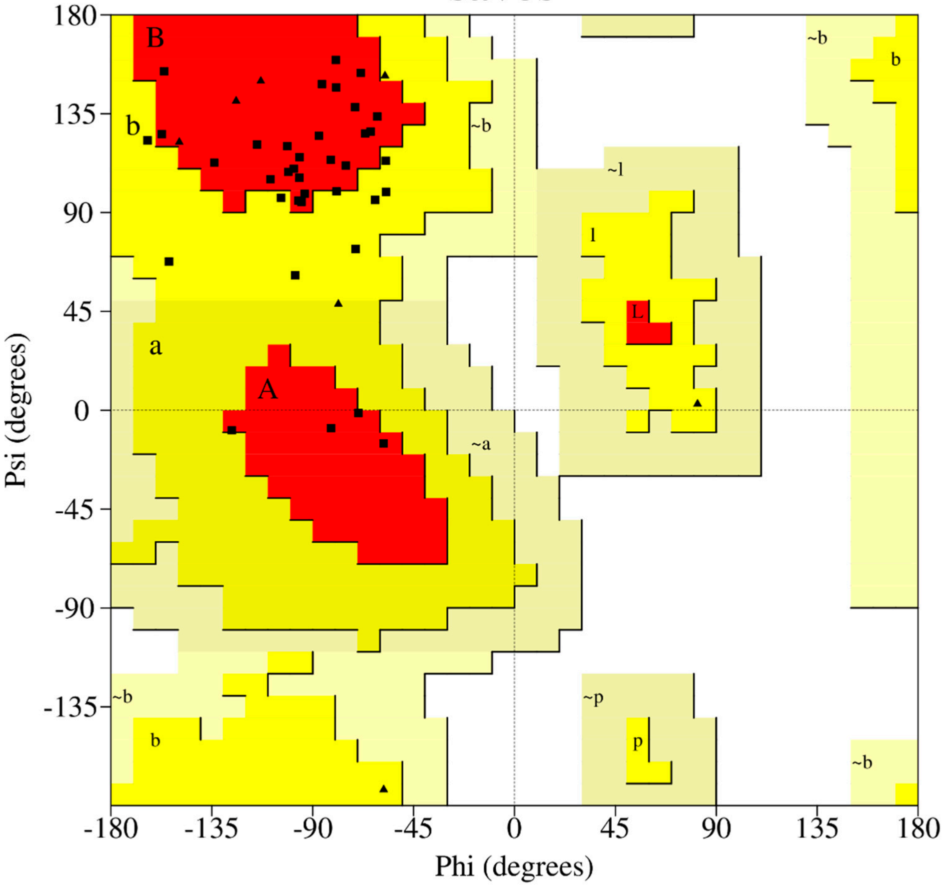

Plot statistics

Residues in most favoured regions [A,B,L]	25	73.5%
Residues in additional allowed regions [a,b,l,p]	9	26.5%
Residues in generously allowed regions [~a,~b,~l,~p]	0	0.0%
Residues in disallowed regions	0	0.0%
	----	------
Number of non-glycine and non-proline residues	34	100.0%
Number of end-residues (excl. Gly and Pro)	1	
Number of glycine residues (shown as triangles)	7	
Number of proline residues	4	

Total number of residues	46	

Based on an analysis of 118 structures of resolution of at least 2.0 Angstroms and R-factor no greater than 20%, a good quality model would be expected to have over 90% in the most favoured regions.

lncRNA2

PROCHECK

Ramachandran Plot

saves

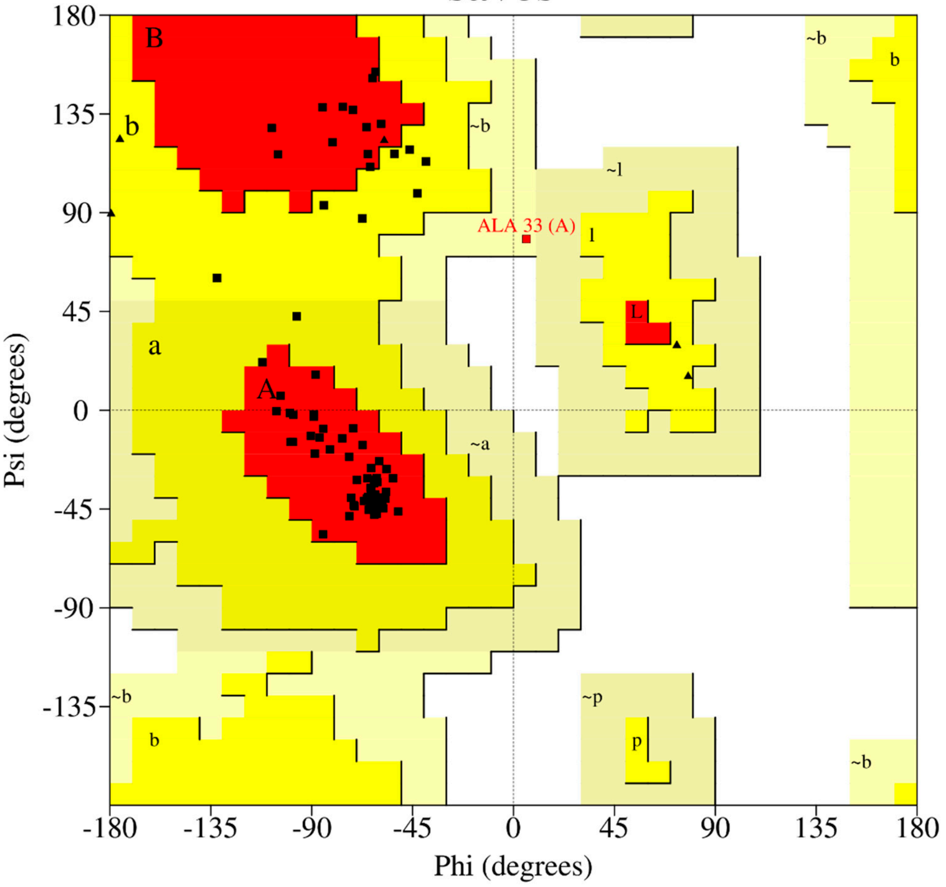

Plot statistics

Residues in most favoured regions [A,B,L]	73	88.0%
Residues in additional allowed regions [a,b,l,p]	9	10.8%
Residues in generously allowed regions [~a,~b,~l,~p]	1	1.2%
Residues in disallowed regions	0	0.0%
	----	------
Number of non-glycine and non-proline residues	83	100.0%
Number of end-residues (excl. Gly and Pro)	2	
Number of glycine residues (shown as triangles)	5	
Number of proline residues	7	

Total number of residues	97	

Based on an analysis of 118 structures of resolution of at least 2.0 Angstroms and R-factor no greater than 20%, a good quality model would be expected to have over 90% in the most favoured regions.

lncRNA3

PROCHECK

Ramachandran Plot

saves

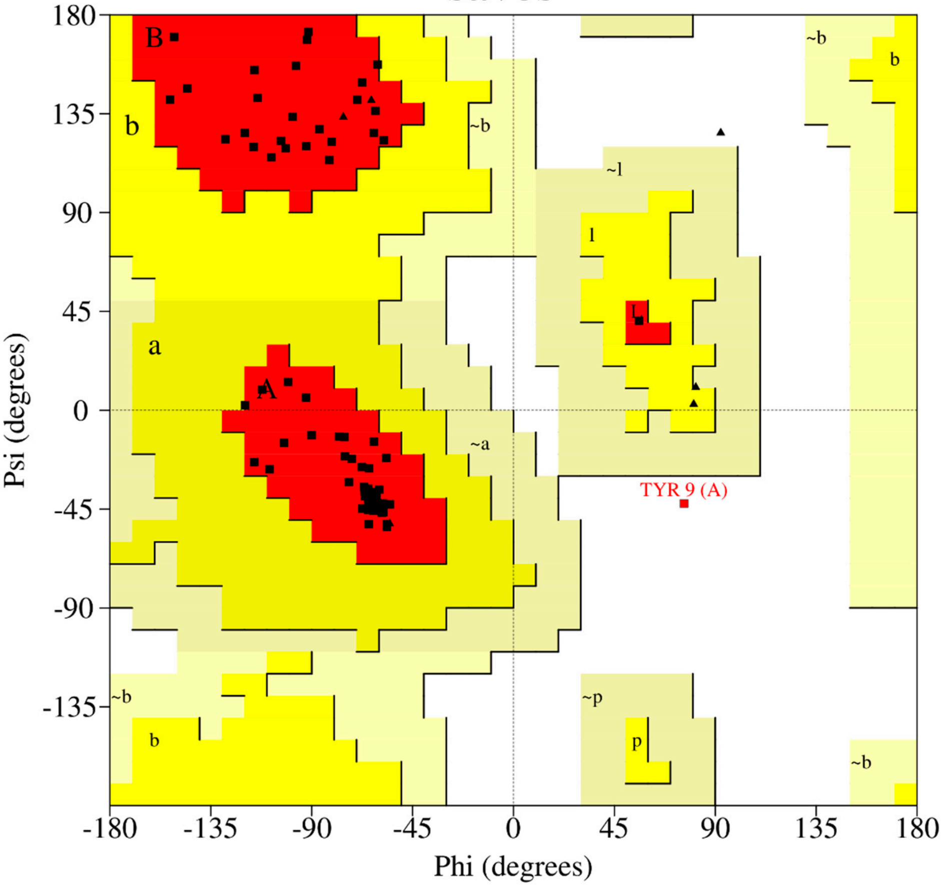

Plot statistics

Residues in most favoured regions [A,B,L]	87	98.9%
Residues in additional allowed regions [a,b,l,p]	0	0.0%
Residues in generously allowed regions [~a,~b,~l,~p]	0	0.0%
Residues in disallowed regions	1	1.1%
	----	------
Number of non-glycine and non-proline residues	88	100.0%
Number of end-residues (excl. Gly and Pro)	2	
Number of glycine residues (shown as triangles)	7	
Number of proline residues	4	

Total number of residues	101	

Based on an analysis of 118 structures of resolution of at least 2.0 Angstroms and R-factor no greater than 20%, a good quality model would be expected to have over 90% in the most favoured regions.

lncRNA4

PROCHECK

Ramachandran Plot

saves

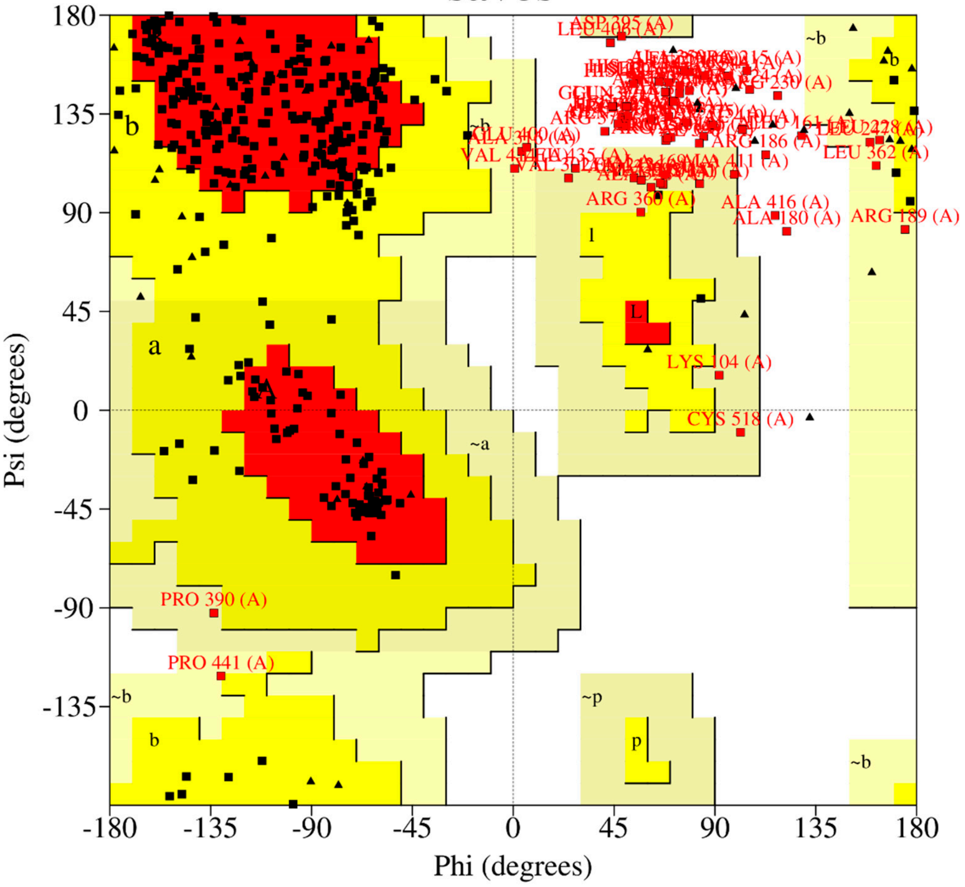

Plot statistics

Residues in most favoured regions [A,B,L]	250	67.6%
Residues in additional allowed regions [a,b,l,p]	59	15.9%
Residues in generously allowed regions [~a,~b,~l,~p]	20	5.4%
Residues in disallowed regions	41	11.1%
	----	------
Number of non-glycine and non-proline residues	370	100.0%
Number of end-residues (excl. Gly and Pro)	2	
Number of glycine residues (shown as triangles)	47	
Number of proline residues	33	

Total number of residues	452	

Based on an analysis of 118 structures of resolution of at least 2.0 Angstroms and R-factor no greater than 20%, a good quality model would be expected to have over 90% in the most favoured regions.

lncRNA5

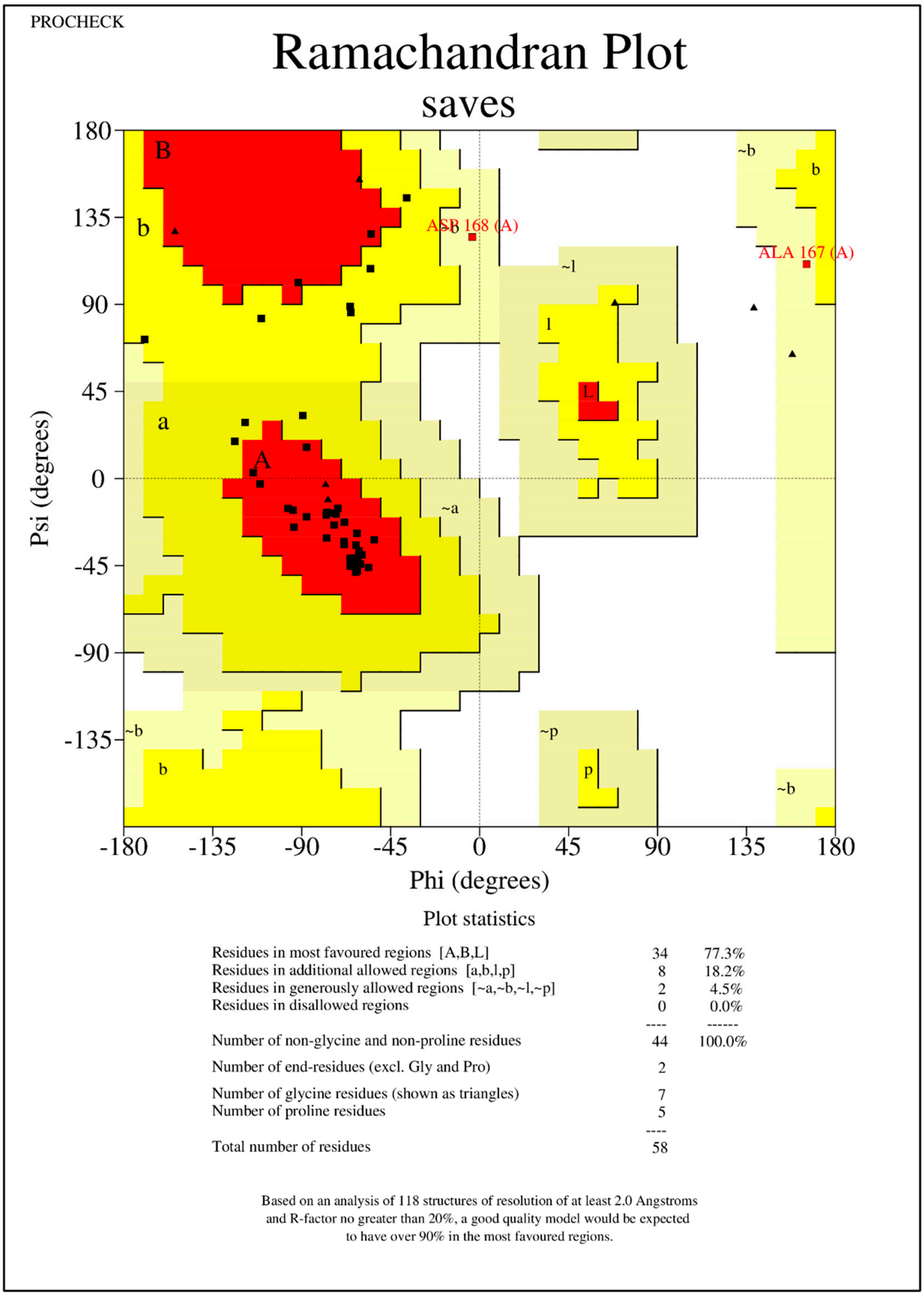
PROCHECK
Ramachandran Plot
saves
180
135
90
45
0
-45
-90
-135
-180
-135
-90
-45
0
45
90
135
180
Psi (degrees)
Phi (degrees)
B
b
A
a
L
l
p
~b
~a
~l
~p
ASP 168 (A)
ALA 167 (A)
Plot statistics
Residues in most favoured regions [A,B,L] 34 77.3%
Residues in additional allowed regions [a,b,l,p] 8 18.2%
Residues in generously allowed regions [~a,~b,~l,~p] 2 4.5%
Residues in disallowed regions 0 0.0%
---- ------
Number of non-glycine and non-proline residues 44 100.0%
Number of end-residues (excl. Gly and Pro) 2
Number of glycine residues (shown as triangles) 7
Number of proline residues 5

Total number of residues 58
Based on an analysis of 118 structures of resolution of at least 2.0 Angstroms
and R-factor no greater than 20%, a good quality model would be expected
to have over 90% in the most favoured regions.

lncRNA7

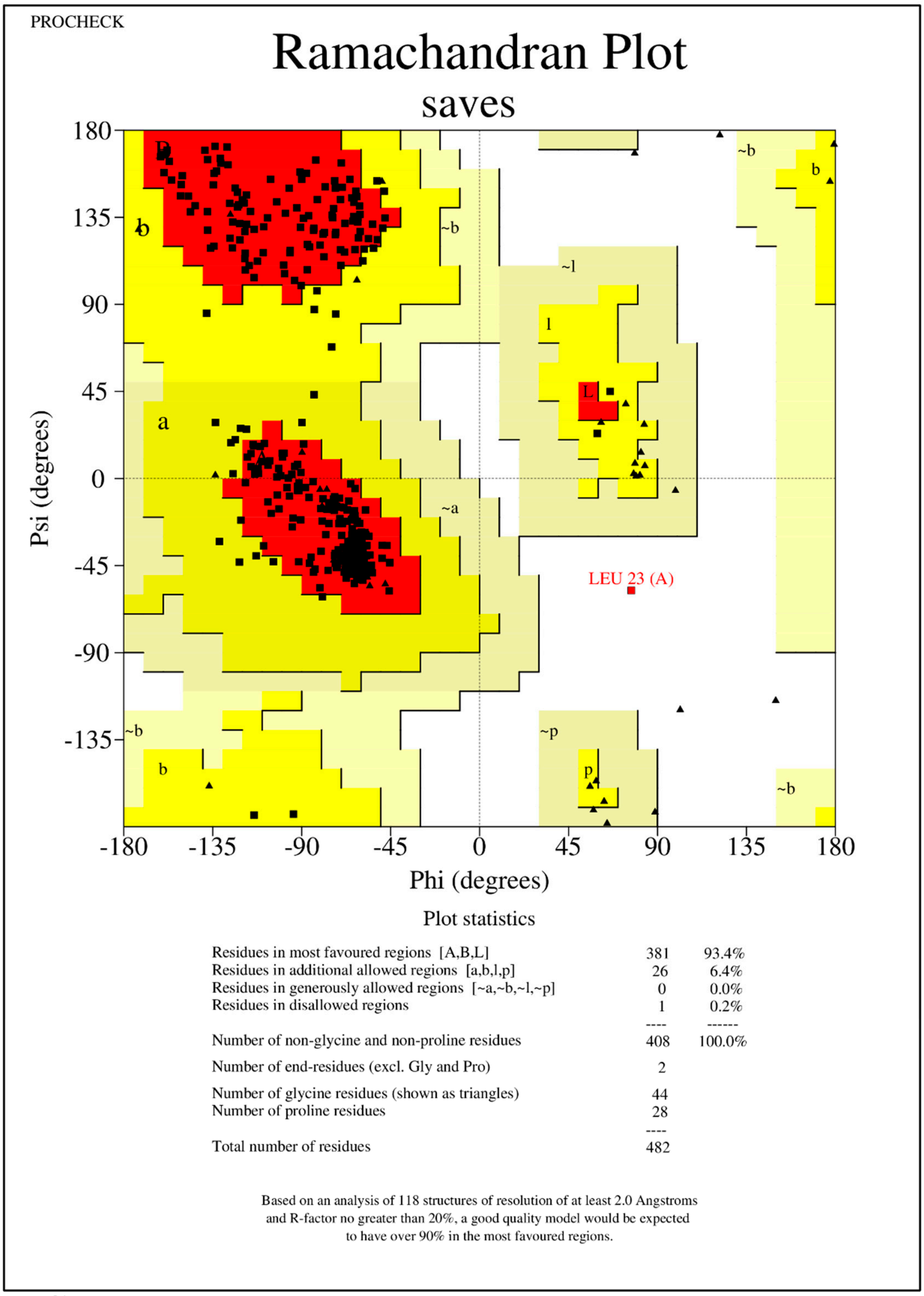

Residues in most favoured regions [A,B,L]	381	93.4%
Residues in additional allowed regions [a,b,l,p]	26	6.4%
Residues in generously allowed regions [~a,~b,~l,~p]	0	0.0%
Residues in disallowed regions	1	0.2%
	----	------
Number of non-glycine and non-proline residues	408	100.0%
Number of end-residues (excl. Gly and Pro)	2	
Number of glycine residues (shown as triangles)	44	
Number of proline residues	28	

Total number of residues	482	

Based on an analysis of 118 structures of resolution of at least 2.0 Angstroms and R-factor no greater than 20%, a good quality model would be expected to have over 90% in the most favoured regions.

lncRNA8

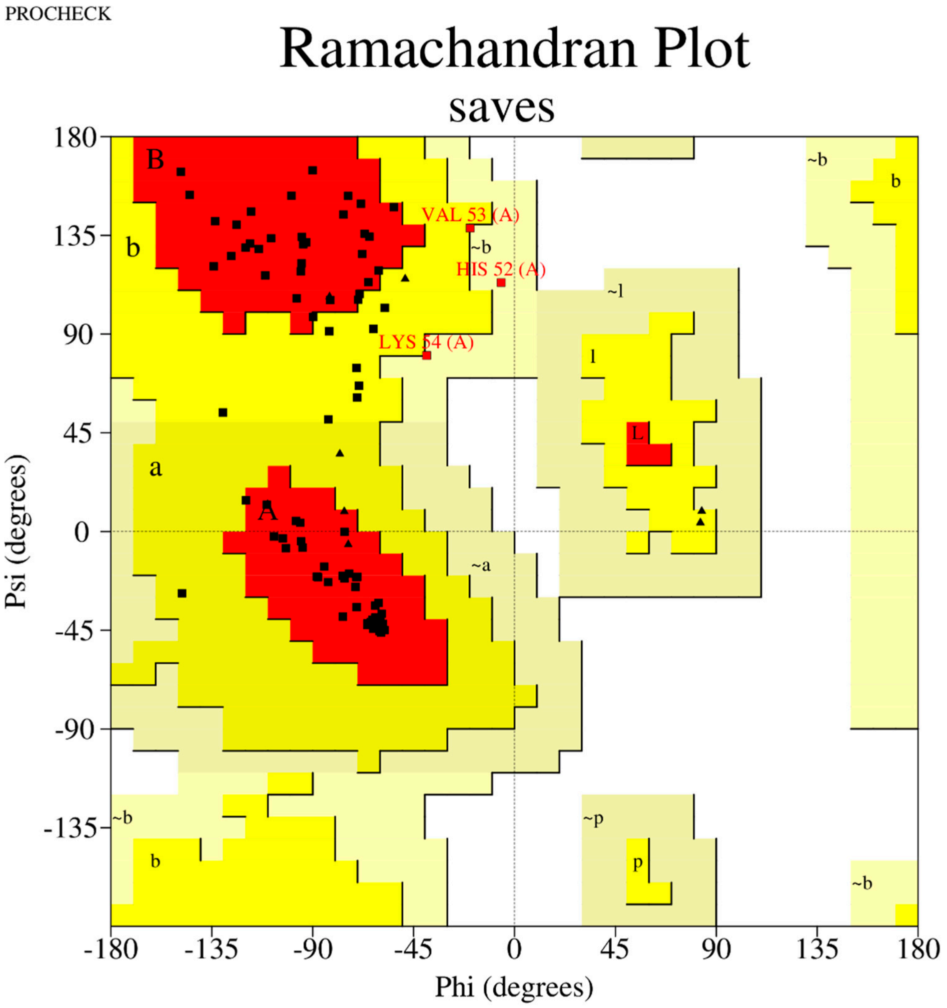

Plot statistics

Residues in most favoured regions [A,B,L]	75	83.3%
Residues in additional allowed regions [a,b,l,p]	12	13.3%
Residues in generously allowed regions [~a,~b,~l,~p]	3	3.3%
Residues in disallowed regions	0	0.0%
	----	------
Number of non-glycine and non-proline residues	90	100.0%
Number of end-residues (excl. Gly and Pro)	1	
Number of glycine residues (shown as triangles)	7	
Number of proline residues	5	

Total number of residues	103	

Based on an analysis of 118 structures of resolution of at least 2.0 Angstroms and R-factor no greater than 20%, a good quality model would be expected to have over 90% in the most favoured regions.

lncRNA11

PROCHECK

Ramachandran Plot

saves

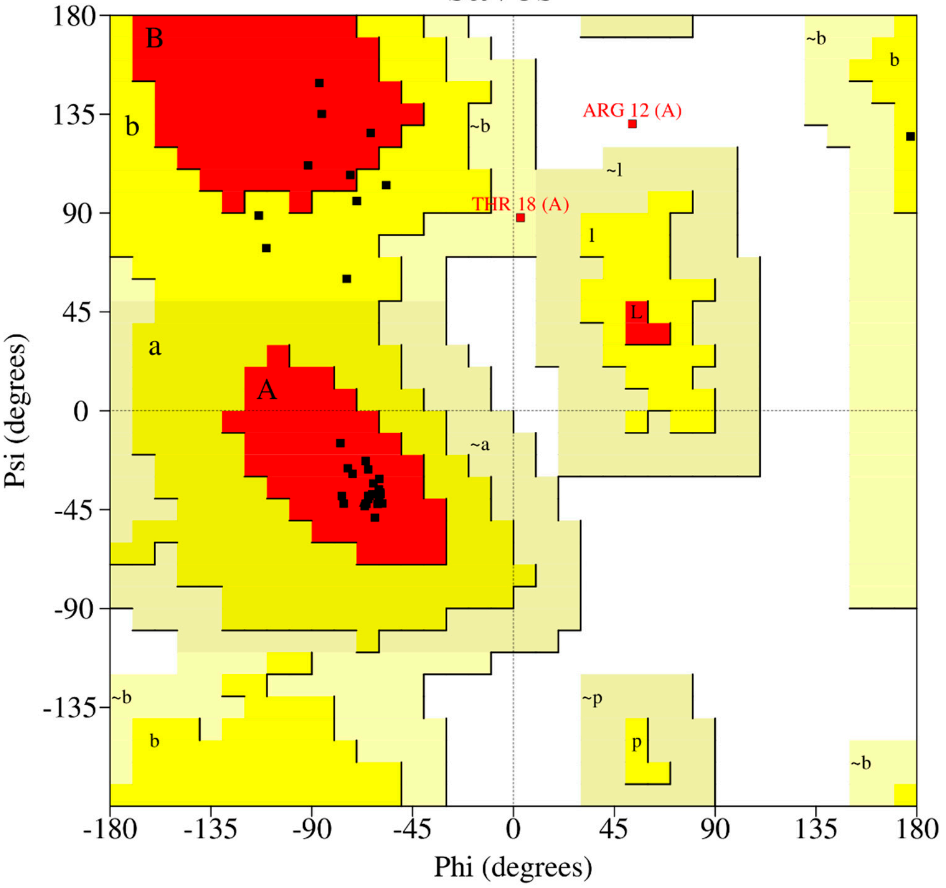

Plot statistics

Residues in most favoured regions [A,B,L]	28	77.8%
Residues in additional allowed regions [a,b,l,p]	6	16.7%
Residues in generously allowed regions [~a,~b,~l,~p]	1	2.8%
Residues in disallowed regions	1	2.8%
	----	------
Number of non-glycine and non-proline residues	36	100.0%
Number of end-residues (excl. Gly and Pro)	2	
Number of glycine residues (shown as triangles)	1	
Number of proline residues	1	

Total number of residues	40	

Based on an analysis of 118 structures of resolution of at least 2.0 Angstroms and R-factor no greater than 20%, a good quality model would be expected to have over 90% in the most favoured regions.

lncRNA12

PROCHECK

Ramachandran Plot

saves

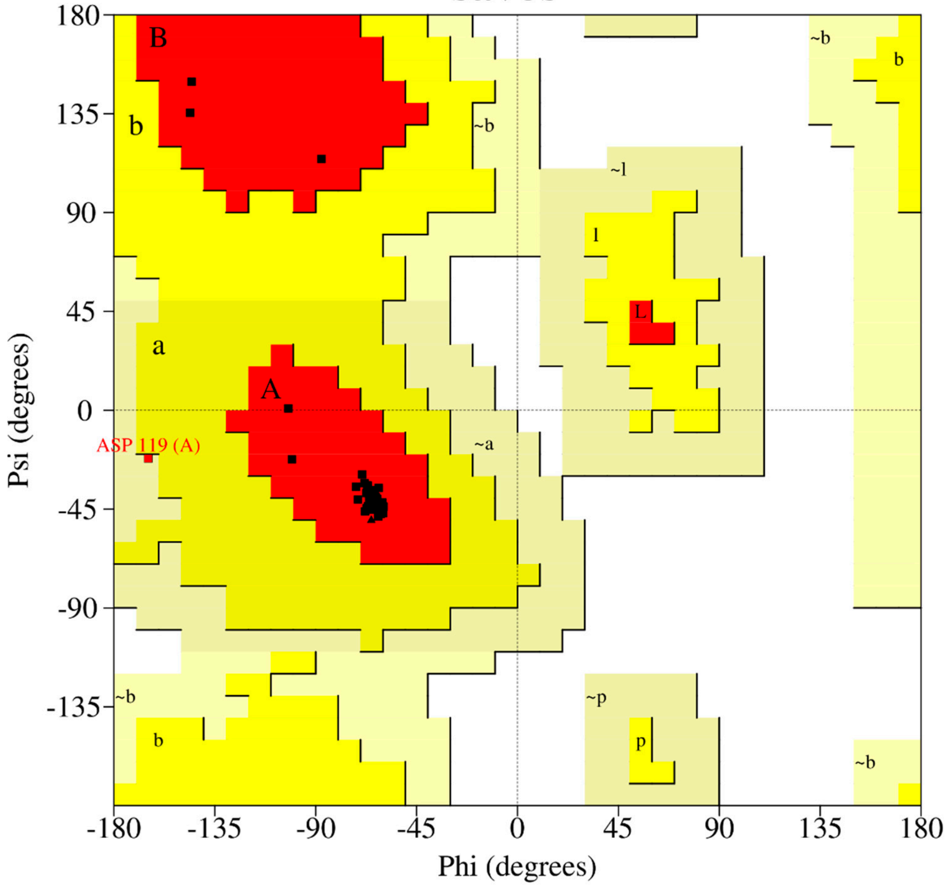

Plot statistics

Residues in most favoured regions [A,B,L]	47	97.9%
Residues in additional allowed regions [a,b,l,p]	0	0.0%
Residues in generously allowed regions [~a,~b,~l,~p]	1	2.1%
Residues in disallowed regions	0	0.0%
	----	------
Number of non-glycine and non-proline residues	48	100.0%
Number of end-residues (excl. Gly and Pro)	2	
Number of glycine residues (shown as triangles)	1	
Number of proline residues	0	

Total number of residues	51	

Based on an analysis of 118 structures of resolution of at least 2.0 Angstroms and R-factor no greater than 20%, a good quality model would be expected to have over 90% in the most favoured regions.

lncRNA13

PROCHECK

Ramachandran Plot

saves

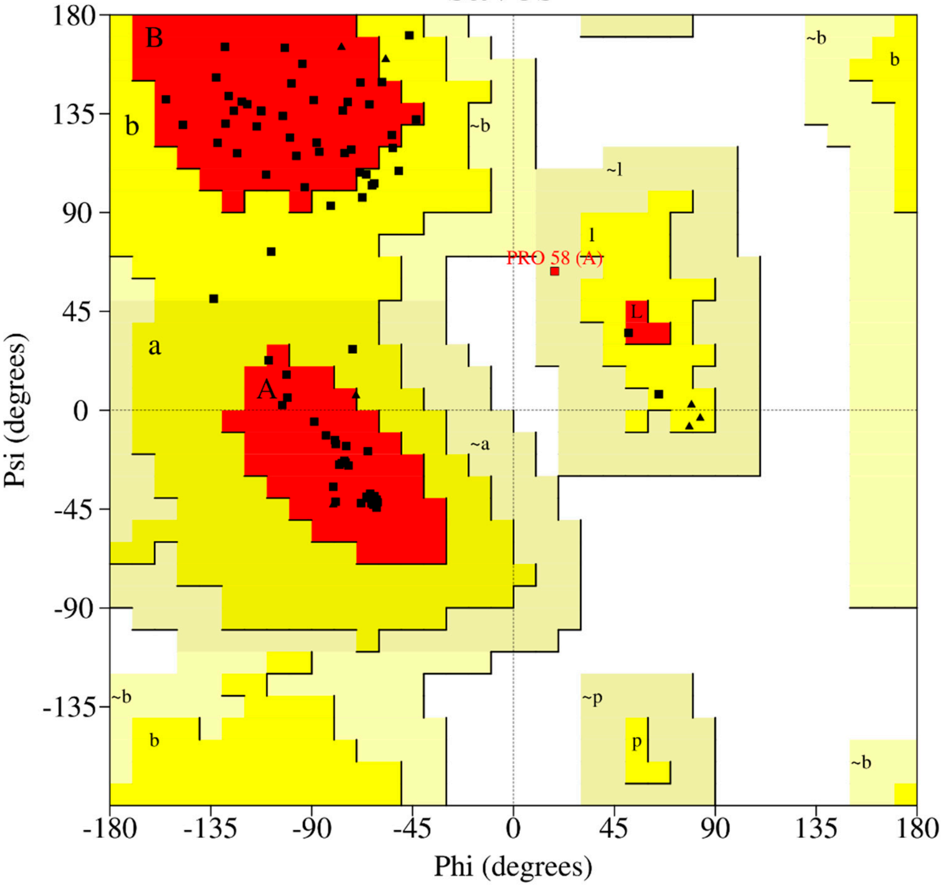

Plot statistics

Residues in most favoured regions [A,B,L]	63	82.9%
Residues in additional allowed regions [a,b,l,p]	13	17.1%
Residues in generously allowed regions [~a,~b,~l,~p]	0	0.0%
Residues in disallowed regions	0	0.0%
	----	------
Number of non-glycine and non-proline residues	76	100.0%
Number of end-residues (excl. Gly and Pro)	2	
Number of glycine residues (shown as triangles)	7	
Number of proline residues	4	

Total number of residues	89	

Based on an analysis of 118 structures of resolution of at least 2.0 Angstroms and R-factor no greater than 20%, a good quality model would be expected to have over 90% in the most favoured regions.

lncRNA14

PROCHECK

Ramachandran Plot

saves

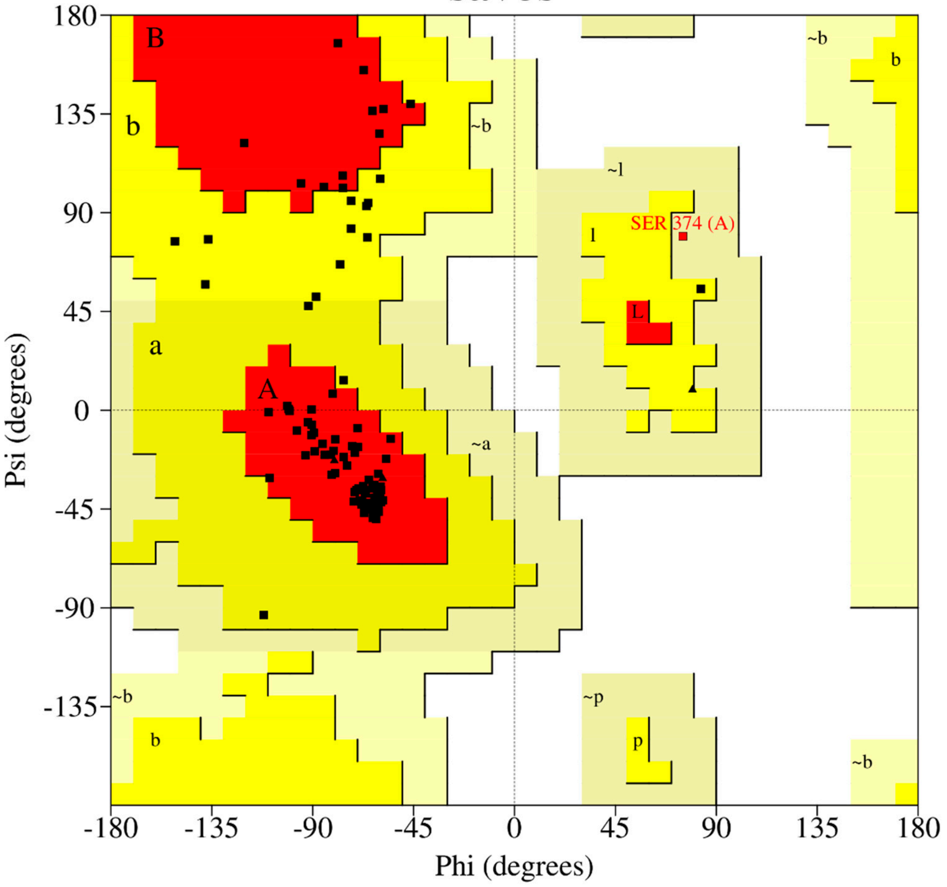

Plot statistics

Residues in most favoured regions [A,B,L]	84	84.8%
Residues in additional allowed regions [a,b,l,p]	14	14.1%
Residues in generously allowed regions [~a,~b,~l,~p]	1	1.0%
Residues in disallowed regions	0	0.0%
	----	------
Number of non-glycine and non-proline residues	99	100.0%
Number of end-residues (excl. Gly and Pro)	2	
Number of glycine residues (shown as triangles)	6	
Number of proline residues	12	

Total number of residues	119	

Based on an analysis of 118 structures of resolution of at least 2.0 Angstroms and R-factor no greater than 20%, a good quality model would be expected to have over 90% in the most favoured regions.

lncRNA15

PROCHECK

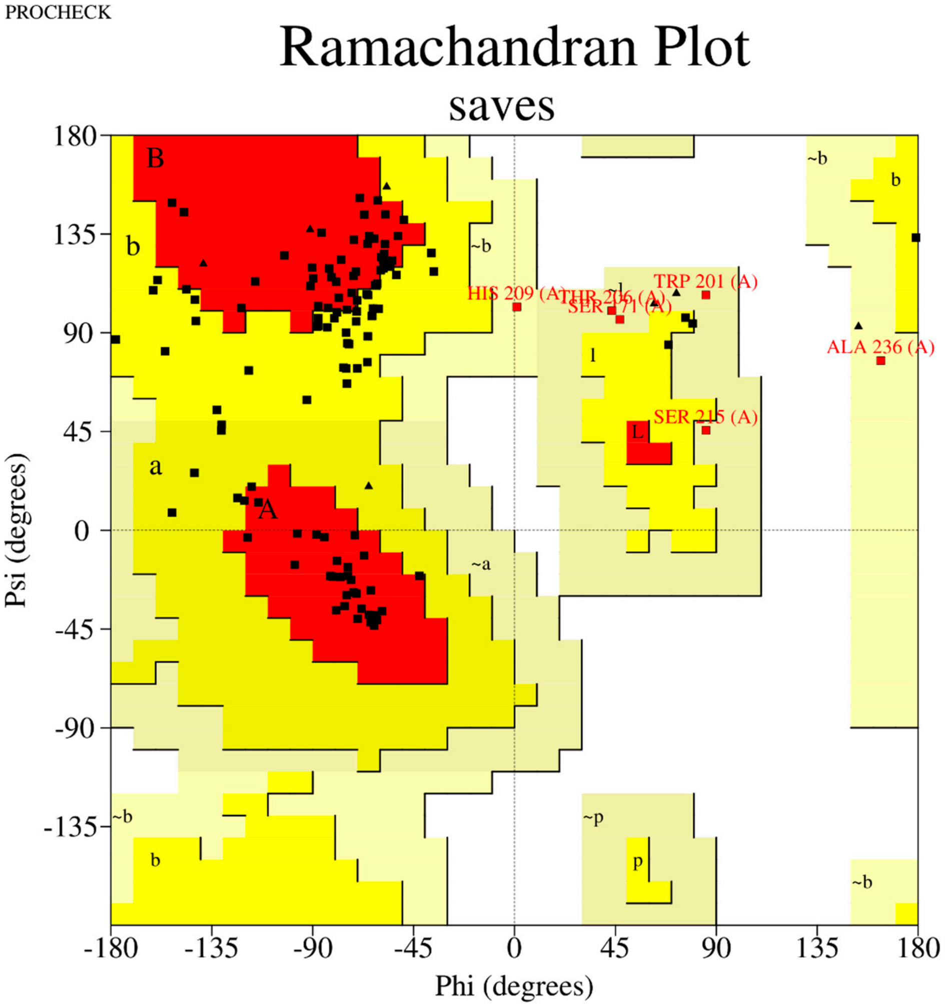

Plot statistics

Residues in most favoured regions [A,B,L]	59	53.2%
Residues in additional allowed regions [a,b,l,p]	46	41.4%
Residues in generously allowed regions [~a,~b,~l,~p]	6	5.4%
Residues in disallowed regions	0	0.0%
	----	------
Number of non-glycine and non-proline residues	111	100.0%
Number of end-residues (excl. Gly and Pro)	2	
Number of glycine residues (shown as triangles)	9	
Number of proline residues	14	

Total number of residues	136	

Based on an analysis of 118 structures of resolution of at least 2.0 Angstroms and R-factor no greater than 20%, a good quality model would be expected to have over 90% in the most favoured regions.

lncRNA16

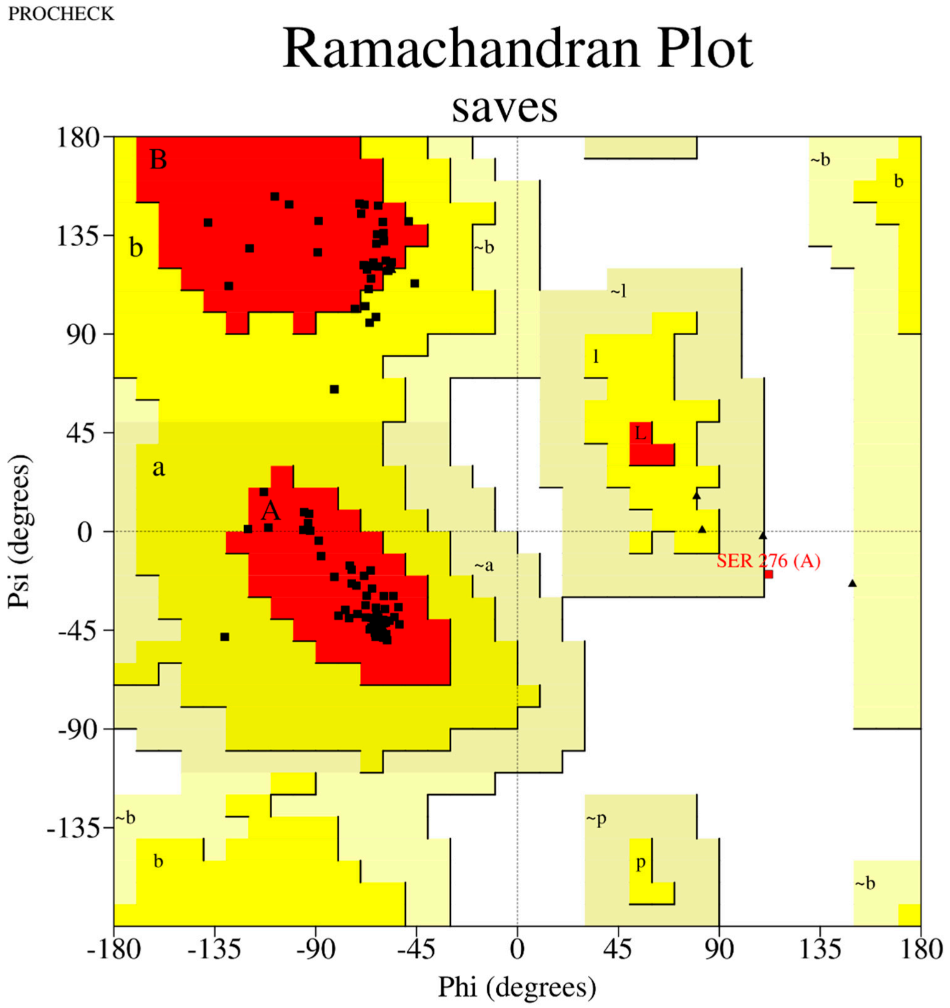

Plot statistics

Residues in most favoured regions [A,B,L]	101	91.8%
Residues in additional allowed regions [a,b,l,p]	8	7.3%
Residues in generously allowed regions [~a,~b,~l,~p]	0	0.0%
Residues in disallowed regions	1	0.9%
	----	------
Number of non-glycine and non-proline residues	110	100.0%
Number of end-residues (excl. Gly and Pro)	2	
Number of glycine residues (shown as triangles)	5	
Number of proline residues	6	

Total number of residues	123	

Based on an analysis of 118 structures of resolution of at least 2.0 Angstroms and R-factor no greater than 20%, a good quality model would be expected to have over 90% in the most favoured regions.

lncRNA17

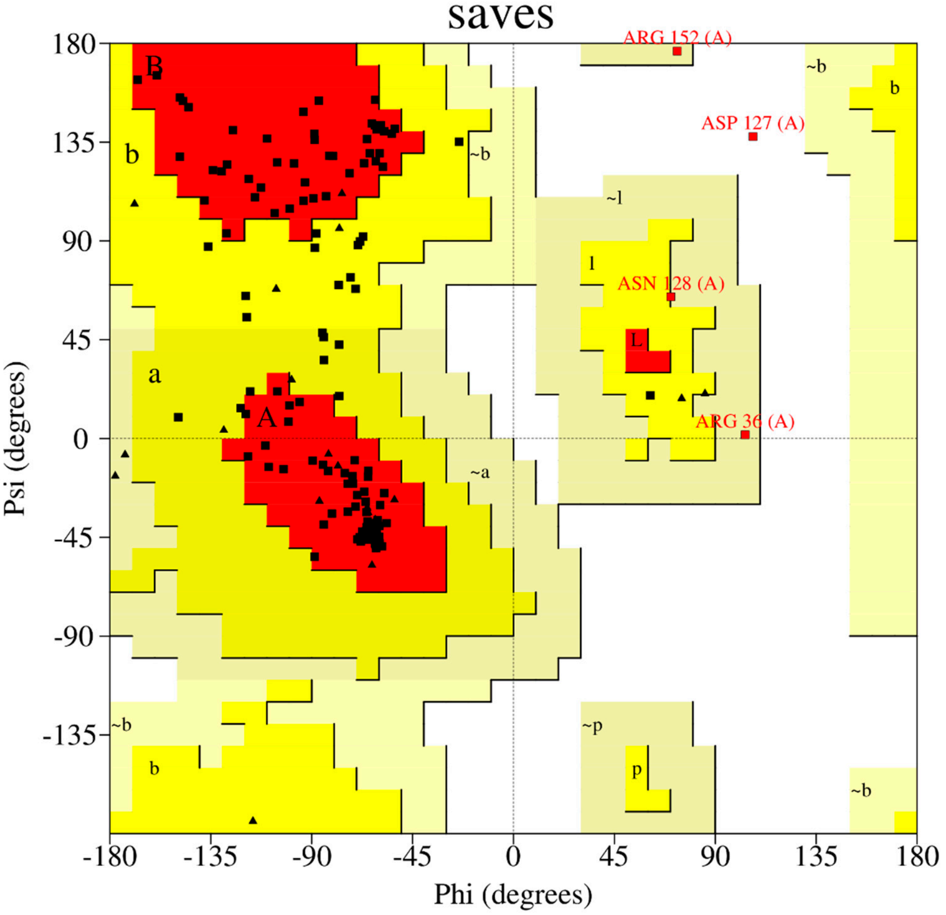

Plot statistics

Residues in most favoured regions [A,B,L]	105	80.8%
Residues in additional allowed regions [a,b,l,p]	21	16.2%
Residues in generously allowed regions [~a,~b,~l,~p]	3	2.3%
Residues in disallowed regions	1	0.8%
	----	------
Number of non-glycine and non-proline residues	130	100.0%
Number of end-residues (excl. Gly and Pro)	2	
Number of glycine residues (shown as triangles)	20	
Number of proline residues	6	

Total number of residues	158	

Based on an analysis of 118 structures of resolution of at least 2.0 Angstroms and R-factor no greater than 20%, a good quality model would be expected to have over 90% in the most favoured regions.

lncRNA18

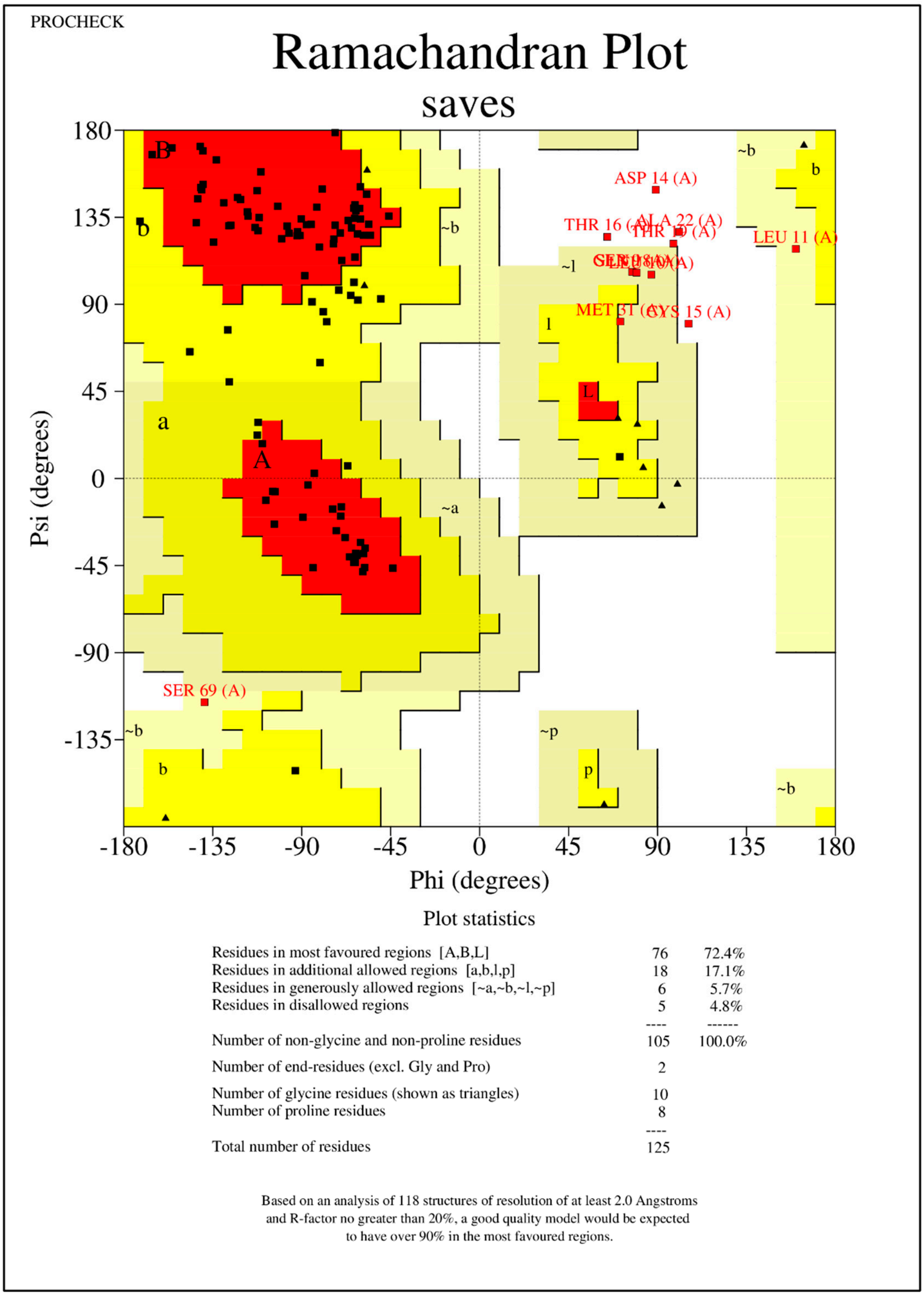
PROCHECK
Ramachandran Plot
saves
Psi (degrees)
Phi (degrees)
ASP 14 (A)
THR 16 (A)
ALA 22 (A)
THR 10 (A)
LEU 11 (A)
MET 31 (A)
CYS 15 (A)
SER 69 (A)
Plot statistics
Residues in most favoured regions [A,B,L] 76 72.4%
Residues in additional allowed regions [a,b,l,p] 18 17.1%
Residues in generously allowed regions [~a,~b,~l,~p] 6 5.7%
Residues in disallowed regions 5 4.8%
Number of non-glycine and non-proline residues 105 100.0%
Number of end-residues (excl. Gly and Pro) 2
Number of glycine residues (shown as triangles) 10
Number of proline residues 8
Total number of residues 125
Based on an analysis of 118 structures of resolution of at least 2.0 Angstroms and R-factor no greater than 20%, a good quality model would be expected to have over 90% in the most favoured regions.

lncRNA19

PROCHECK

Ramachandran Plot

saves

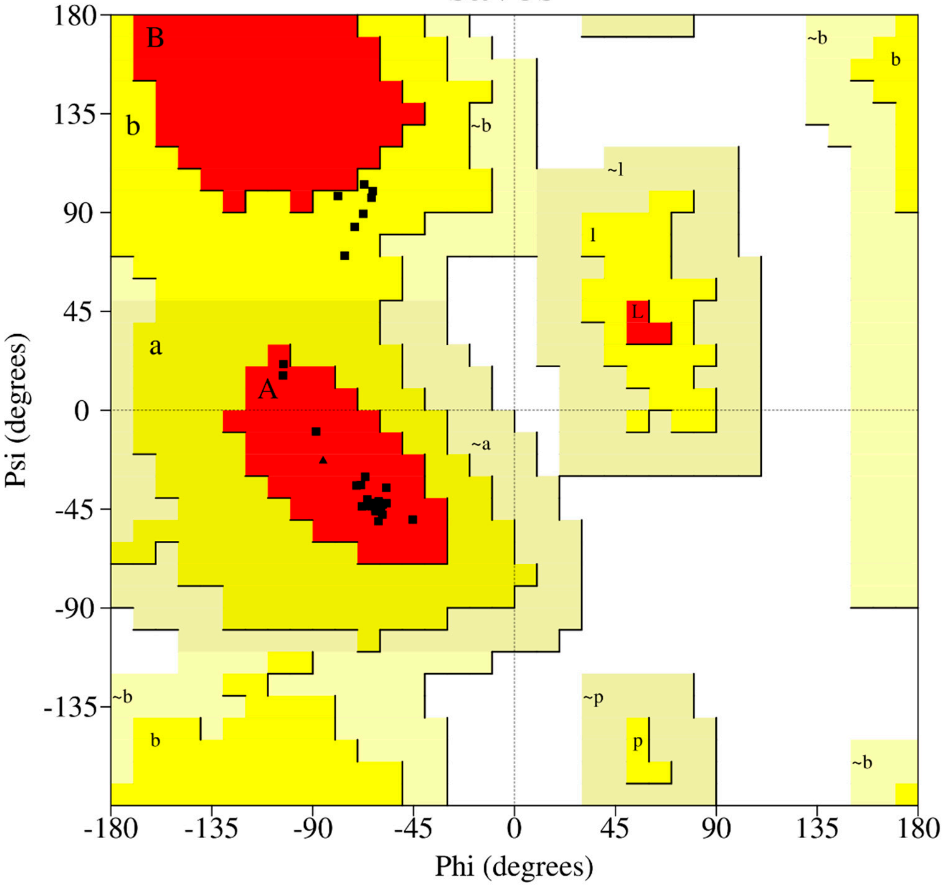

Plot statistics

Residues in most favoured regions [A,B,L]	31	81.6%
Residues in additional allowed regions [a,b,l,p]	7	18.4%
Residues in generously allowed regions [~a,~b,~l,~p]	0	0.0%
Residues in disallowed regions	0	0.0%
	----	------
Number of non-glycine and non-proline residues	38	100.0%
Number of end-residues (excl. Gly and Pro)	2	
Number of glycine residues (shown as triangles)	1	
Number of proline residues	1	

Total number of residues	42	

Based on an analysis of 118 structures of resolution of at least 2.0 Angstroms and R-factor no greater than 20%, a good quality model would be expected to have over 90% in the most favoured regions.

lncRNA20

PROCHECK

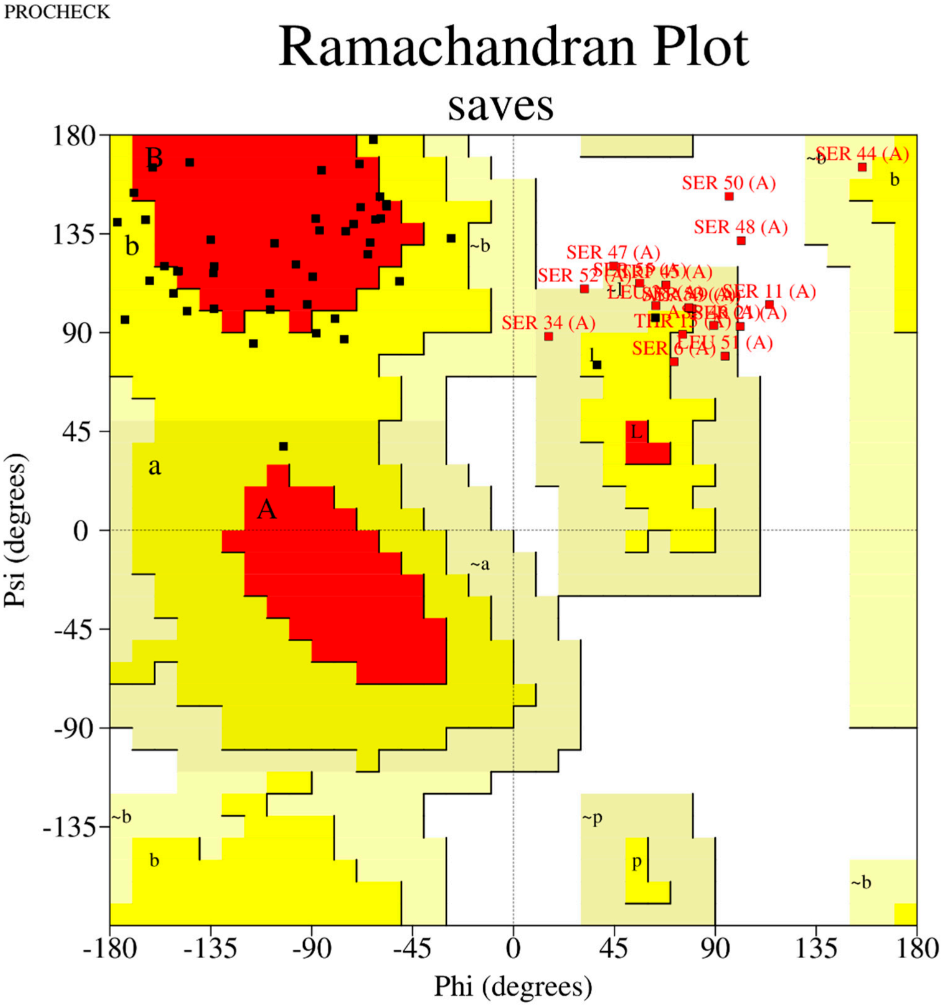

Plot statistics

Residues in most favoured regions [A,B,L]	25	43.9%
Residues in additional allowed regions [a,b,l,p]	15	26.3%
Residues in generously allowed regions [~a,~b,~l,~p]	12	21.1%
Residues in disallowed regions	5	8.8%
	----	------
Number of non-glycine and non-proline residues	57	100.0%
Number of end-residues (excl. Gly and Pro)	2	
Number of glycine residues (shown as triangles)	0	
Number of proline residues	6	

Total number of residues	65	

Based on an analysis of 118 structures of resolution of at least 2.0 Angstroms and R-factor no greater than 20%, a good quality model would be expected to have over 90% in the most favoured regions.

lncRNA21

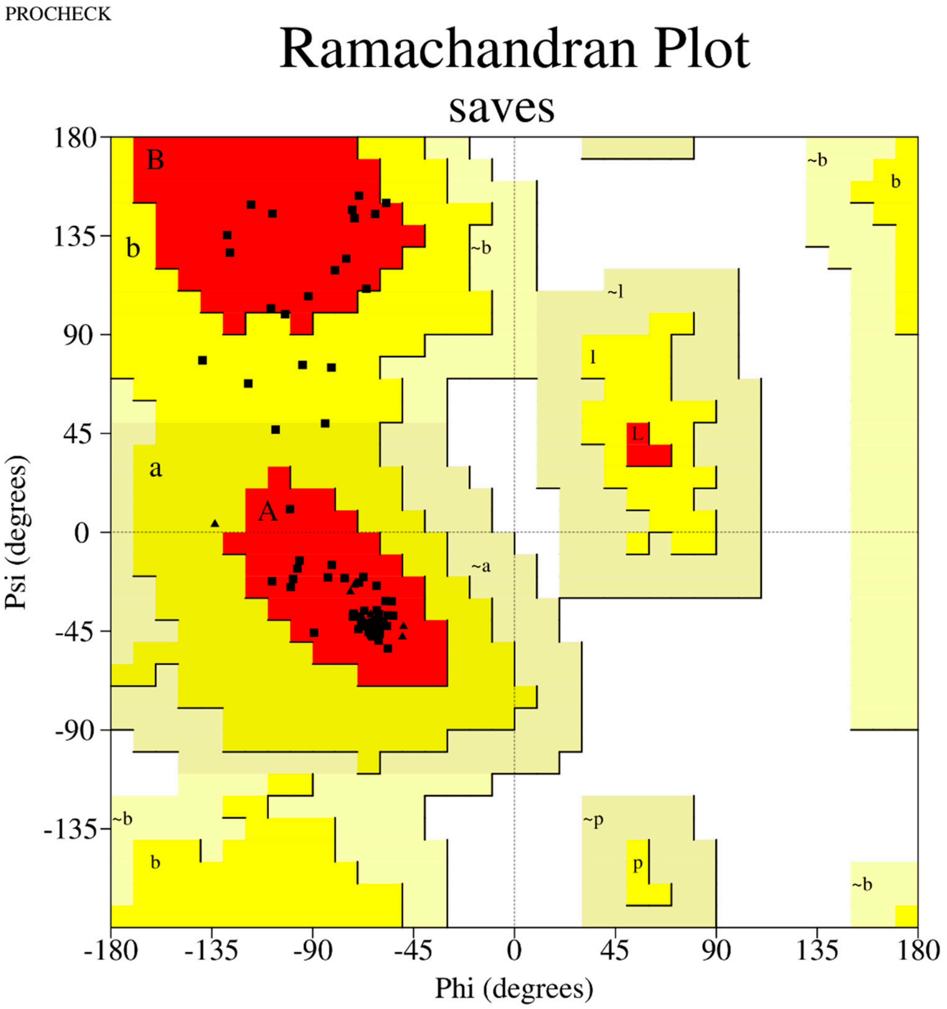

Plot statistics

Residues in most favoured regions [A,B,L]	68	91.9%
Residues in additional allowed regions [a,b,l,p]	6	8.1%
Residues in generously allowed regions [~a,~b,~l,~p]	0	0.0%
Residues in disallowed regions	0	0.0%
	----	------
Number of non-glycine and non-proline residues	74	100.0%
Number of end-residues (excl. Gly and Pro)	2	
Number of glycine residues (shown as triangles)	8	
Number of proline residues	6	

Total number of residues	90	

Based on an analysis of 118 structures of resolution of at least 2.0 Angstroms and R-factor no greater than 20%, a good quality model would be expected to have over 90% in the most favoured regions.

lncRNA22
